# Parametric investigation of rectangular CFRP-confined concrete columns reinforced by inner elliptical steel tubes using finite element and machine learning models

**DOI:** 10.1016/j.heliyon.2023.e23666

**Published:** 2023-12-15

**Authors:** Haytham F. Isleem, Besukal Befikadu Zewudie, Alireza Bahrami, Rakesh Kumar, Wang Xingchong, Pijush Samui

**Affiliations:** a School of Applied Technologies, Qujing Normal University, Qujing 655011, Yunnan, China; b Department of Building Engineering, Energy Systems and Sustainability Science, Faculty of Engineering and Sustainable Development, University of Gävle, 801 76 Gävle, Sweden; c Faculty of Civil and Environmental Engineering, Jimma Institute of Technology, Jimma University, Ethiopia; d Department of Civil Engineering, National Institute of Technology Patna, India

**Keywords:** Confined concrete, Steel tube, Dilation angle, Lateral strain, Strength enhancement, Machine learning

## Abstract

Nowadays, due to the structural advantages gained by combining three different materials' properties, columns made of carbon-fiber reinforced polymer (CFRP)-confined concrete with inner steel tube have received researchers' interest. This article presents the nonlinear finite element analysis and multiple machine learning (ML) model-based study on the behavior of round corner rectangular CFRP-confined concrete short columns reinforced by the inner high-strength elliptical steel tube under the axial load. The reliability of the proposed nonlinear finite element model was verified against the existing experimental investigations. The effects of the parameters such as the concrete grade, thickness of reinforcing steel tube, cross-sectional size of inner steel tube, and thickness of CFRP on the behavior of the columns are comprehended in this study. Furthermore, multiple ML models were proposed to predict the ultimate axial load, ultimate axial strain, and lateral strain of the test specimens. The reliability of the proposed ML models was evaluated by six distinct performance metrics. From the parametric investigation, it was found that concrete with lower compressive strength gained more strength enhancement because of confinement between CFRP and the inner steel tube than high-strength concrete relative to its unconfined compressive strength. The proposed ML models of extreme gradient boosting and random forest provided the best-fit results than the artificial neural network and Gaussian process regression models in predicting the axial load and axial and lateral strains of the columns.

## Highlights

• Compressive behavior of CFRP-confined-steel reinforced rectangular concrete columns.
• Finite Element Model (FEM) Development.
• Parametric Analysis.
• Study of Test Variables Effects.
• Machine Learning (ML) Model Development.

## Notations

$2a_{o}$: outer width of elliptical steel tube (mm)
$2b_{o}$: outer depth of elliptical steel tube (mm)
*b*: outer width of CFRP (mm)
*h*: outer depth of CFRP (mm)
$E_{frp}$: elastic modulus of CFRP (MPa)
$t_{frp}$: thickness of CFRP (mm)
$f_{y}$: yield strength of steel tube (MPa)
$f_{u}$: ultimate strength of steel tube (MPa)
$t_{s}$: thickness of steel tube (mm)
$P_{m}$: mean perimeter of elliptical steel tube (mm)
$A_{e,st}$: cross-sectional area of elliptical steel tube (mm^2^)
$A_{nc}$: nominal cross-sectional area of concrete (mm^2^)
$f_{r}$: residual stress (MPa)
$r_{c}$: radius of corner section (mm)
$f_{cc}$: ultimate confined concrete strength (MPa)
$\varepsilon_{cc}$: confined concrete strain at ultimate strength (mm/mm)
$\varepsilon_{co}$: ultimate unconfined concrete strain of standard cylinder (mm/mm)
$f_{co}$: ultimate unconfined concrete strength of standard cylinder (MPa)
$f_{cc}/f_{co}$: enhancement ratio of confined concrete strength
$\varepsilon_{cc}/\varepsilon_{co}$: enhancement ratio of confined concrete strain
ANN: artificial neural network
CFFT: concrete-filled CFRP tube
CFRP: carbon-fiber reinforced polymer
DSTCC: double skin tube concrete column
FEA: finite element analysis
GPR: Gaussian process regression
RF: random forest
XGB: extreme gradient boosting

## 1 Introduction

Several engineering techniques are employed to enhance the strength, durability, and weather resistance of concrete structures at present. Confining concrete using fiber-reinforced polymer (FRP) is among the widely practiced structural engineering techniques to alleviate the brittle nature and durability of concrete [1]. Confining concrete between external FRP and the inner steel tube to form double skin tube concrete columns (DSTCCs) offers numerous advantages such as the external FRP tube serves as the formwork for casting concrete resulting in a reduction of the cost required for concrete molding formwork. Owing to high corrosion resistance nature of FRP, FRP-confined structures are suitable in harsh environmental conditions. The effective confinement from the external FRP tube and inner steel tube improves the ductility and energy dissipation of DSTCCs. This behavior alleviates the performance of DSTCCs in seismic regions [[1], [2], [3], [4], [5], [6], [7], [8], [9], [10], [11], [12], [13], [14], [15]]. The infilled concrete in DSTCCs suppresses the early local buckling of the inner steel tube and optimizes the load-carrying capacity of the inner steel tube [9]. These structural advantages gained by combining FRP, concrete, and steel tube to form DSTCCs have attracted the attention of researchers.

The experimental investigation reported by Teng et al. [16] demonstrated the advantages gained by combining the three constituents of materials in forming FRP–concrete–steel tube columns. Since then, the experimental research works [1,2,4,10,17,18] revealed the effects of different parameters on the behavioral responses of these columns with various shapes under concentric and eccentric compression static loads.

The confinement effectiveness and behavior of DSTCCs and FRP tube-filled concrete columns depend on several factors. The strength and strain enhancements and energy absorption of FRP-confined concrete decrease remarkably with the increase of the unconfined concrete strength [14]. The impact of the FRP thickness, FRP ply configurations, and aspect ratio on the strength and strain behavior of FRP-confined columns was reported by Refs. [[19], [20], [21], [22], [23], [24]].

Gu et al. [25] indicated the effects of the FRP fracture strain on the drift capacity of FRP-retrofitted reinforced concrete under simulated seismic loads. Under the monotonic compression load, circular carbon-fiber reinforced polymer (CFRP)-confined concrete shows better confinement effectiveness than rectangular CFRP-confined concrete [26]. The unconfined concrete represents brittle behavior after reaching the ultimate strength, while the confining FRP improves the infilled concrete strength and allows the confined concrete to exhibit ductile behavior [17,18]. Therefore, due to the extra strength gained from confining FRP, the stress-strain response of the confined concrete is different from that of the unconfined concrete. So far, several researchers proposed multiple stress-strain response models of the confined concrete under static compression and simulated seismic loads [[28], [29], [30], [31], [32], [33], [34], [35], [36], [37], [38], [39], [40], [41]].

**Fig. 1:**
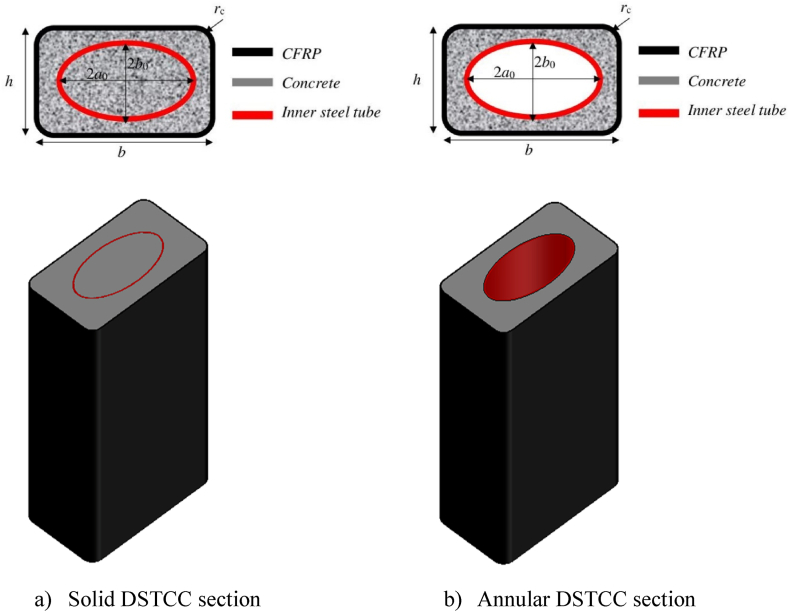
Cross-sectional detail of DSTCCs with inner elliptical steel tube.

However, most of the aforementioned studies describe the structural behavior of FRP-confined to either plain concrete or reinforced concrete columns. But encasing the high-strength steel tube in FRP-confined plain concrete to form DSTCCs, as displayed in Fig. 1, improves the strength and strain.

**Fig. 2:**
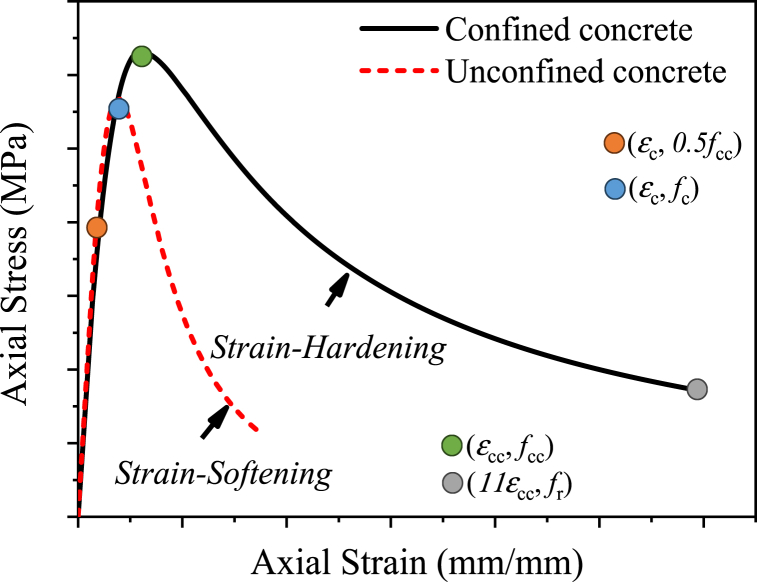
Stress-strain curves of uniaxial confined and unconfined concrete.

The existing literature on DSTCCs can be grouped into two types: 1) DSTCCs in which the inner steel tube is left void (annular DSTCCs), as illustrated in Fig. 1 (b), and 2) DSTCCs in which the inner steel tube is also filled with concrete (solid DSTCCs), as depicted in Fig. 1 (a). Most of the studies on DSTCCs emphasize annular DSTCCs [2,16,19,42,[43], [44]]. However, there is still a gap to be addressed in the behavior of solid DSTCCs. The experimental tests [1,45] justified the effects of the FRP thickness and aspect ratio on circular and rectangular solid DSTCCs.

However, the impacts of the parameters such as the strength of the unconfined concrete and the thickness and size of the inner steel tube on the strength and structural response of solid DSTCCs are not fully comprehended. Therefore, this article presents a nonlinear finite element analysis (FEA) of short rectangular solid DSTCCs. The developed test models are limited to short rectangular columns of CFRP-confined concrete with cylindrical unconfined compressive strength in the range between 30 MPa and 60 MPa, which are reinforced by the high-strength elliptical steel tube. The parametric study was conducted on models with the aspect ratio in the range of 1.0 – 2.5, and the thickness of the inner steel tube was limited in the range of 3 mm – 8 mm.

The reliability of the proposed finite element model (FEM) was verified by comparing its results to relevant experimental test results provided by Refs. [1,45]. Parametric study was done to reveal the effects of the concrete strength and thickness and size of inner elliptical steel tube on the performance and structural response of short round corner rectangular solid DSTCCs. Moreover, multiple machine learning (ML) models were proposed for predicting the ultimate axial load and the axial and lateral strains of DSTCCs. The accuracy of the developed ML models in predicting the load-strain relationship of DSTCCs under the concentric axial load was evaluated using multiple performance metrics.

## 2 Finite element modeling and analysis program

In this study, the finite element software ABAQUS was utilized. In this section, the non-linear behavior of each constituent materials used in modeling DSTCCs is discussed. Also, detail of the mesh size, elemental type, analysis step, boundary conditions, and object interaction considered in FEA is presented.

### 2.1 Properties of materials

Linear and nonlinear material behavior was used in defining the behavior of materials in FEA. The detail of each material behavior in DSTCCs is described in the following subsections.

#### 2.1.1 Modeling behavior of CFRP

In this study, a unidirectional CFRP with a nominal thickness of 0.167 mm and density of 1.7 e^−^^9^ g/mm^3^ was employed. The material behavior of CFRP was idealized as orthotropic linear elastic up-to-tensile rupture. In ABAQUS, the elastic material behavior of CFRP was modeled by defining its engineering constants such as the elastic modulus (*E*, MPa), Poisson's ratio (ν), and shear modulus (*G*, MPa) in the material's principal direction. The constants were adopted from the existing literature, Ref. [46], as given in Table 1.

**Table 1:** Engineering constants of CFRP [46].

$E_{11}$ (MPa)	$E_{22}$ (MPa)	$E_{33}$ (MPa)	$\upsilon_{12}$	$\upsilon_{13}$	$\upsilon_{23}$	$G_{12}$ (MPa)	$G_{13}$ (MPa)	$G_{23}$ (MPa)
234900	2900	2900	0.26	0.28	0.26	3920	1130	3920

#### 2.1.2 Material property of confined concrete

Unconfined concrete demonstrates brittle behavior under uniaxial monotonic loading after attaining the ultimate strength. Confining concrete using steel tube or composite FRP improves the behavior of concrete and allows concrete to perform as an inelastic (elastic-plastic) material [47]. The strength and strain enhancements of the confined concrete mainly depend on the degree of confinement provided [47]. The core concrete confined in CFRP acquires the confinement's pressure as the applied axial load increases and concrete starts expanding laterally. This action of confinement is passive interaction. In FEA, modeling the behavior of concrete stress-strain (σ−ε) relation by taking into account this confinement pressure plays a vital role in the accuracy of the analysis results.

Several researchers have proposed different stress-strain (σ−ε) relation models of the confined concrete that can be used in modeling FRP and the steel tube's confined concrete behavior in FEA [[35], [36], [37], [38]]. The stress-strain relation of the confined concrete does not only depend on the concrete strength grade and confinement stress, as generalized in this subsection. The history of the development of strain in the confining material, concrete strength, composition of concrete, and micro-structure and wet packing density of concrete also has effects on the stress-strain relation of the confined concrete [[48], [49], [50], [51], [52], [53], [54]]. In this research, the three-stage stress-strain (σ−ε) relation of the confined concrete proposed by Hu and Schnobrich [55] was modified in modeling the behavior of CFRP-confined concrete. As shown in Fig. 2, the proposed stress-strain relation for the confined concrete consists of three regions, linear elastic region, strain hardening region, and strain softening region. It should be noted that at the early loading, the effect of confinement is insignificant for the ascending curve, the linear stress-strain curve of the unconfined concrete is appropriate in modeling the confined concrete in the linear elastic region [56], and the confined concrete stress at the proportional limit point is assumed to be half of the ultimate confined concrete strength ($f_{cc})$. After the proportional limit point, due to the tri-axial stress from confinement, the confined concrete exhibits strain-hardening plasticity. In this study, for the strain hardening region of the ascending curve, Eq. (1) from Ref. [57] was used alongside the recommended values of the stress ratio ($R_{\sigma }$) and strain ratio ($R_{\varepsilon }$) from Ref. [55] in which both were 4.

(1) $$f_{cc}=\frac{E_{cc}\varepsilon }{1+\left(R+R_{E}-2\right)\left(\frac{\varepsilon }{\varepsilon_{cc}}\right)-\left(2R-1\right){\left(\frac{\varepsilon }{\varepsilon_{cc}}\right)}^{2}+R{\left(\frac{\varepsilon }{\varepsilon_{cc}}\right)}^{3}}$$

where $f_{cc}$ (MPa) and ε (mm/mm) are the strength and strain of the confined concrete in the hardening region, respectively. *R* and *R*_*E*_ of Eq. (1) were determined from Eqs. (2) and (3), respectively.

(2) $$R=\frac{R_{E}\left(R_{\sigma }-1\right)}{{\left(R_{\varepsilon }-1\right)}^{2}}-\frac{1}{R_{\varepsilon }}$$

(3) $$R_{E}=\frac{E_{cc}\varepsilon_{cc}}{f_{cc}}$$

The axial strain ($\varepsilon_{cc})$ of the confined concrete associated with the ultimate strength of the confined concrete ($f_{cc}$) was obtained by utilizing Eq. (4) derived by Nicolo et al. [58] in which $f_{c}$ is the unconfined concrete strength in MPa.

(4) $$\varepsilon_{cc}=0.00076+\sqrt{\left(0.626f_{c}-4.33\right)\times 10^{-7}}$$

In the elastic range, the elastic modulus ($E_{cc}$, MPa) of the confined concrete was calculated using Eq. (5) recommended by ACI [59]. Poisson's ratio of concrete was set to 0.2.

(5) $$E_{cc}=4730\sqrt{f_{cc}}$$

where $f_{cc}$ is the ultimate strength of the confined concrete in MPa.

Due to the induced tri-axial stress from CFRP or steel tube confinement, the confined concrete undergoes the strain-softening region unlike the unconfined concrete. In this study, for modeling the stress-strain relation of the confined concrete in the strain–softening descending region, Eq. (6) proposed by Han et al. [60] was employed with $\beta_{o}=0.6$ and $\eta_{}$ = 2.

(6) $$f_{2}=\frac{\left(\frac{\varepsilon }{\varepsilon_{cc}}\right)f_{c}}{\beta_{o}{\left(\frac{\varepsilon }{\varepsilon_{cc}}-1\right)}^{\eta }+\left(\frac{\varepsilon }{\varepsilon_{cc}}\right)}\;\;\;\;\;\;\;\;\;\;\;\;\;\;\;\;\;\;\;\;\;\;\;\;\;\;\;\;\;\;\varepsilon >\varepsilon_{cc}$$

where $f_{2}$ is the compressive strength of the confined concrete in MPa.

The residual stress ($f_{r}$, MPa) after the rupture of the confined concrete was determined from the descending curve at the recommended strain value of $11\varepsilon_{cc}$.

ABAQUS offers several models for defining the behavior of elastic-plastic materials like the confined concrete. In the current study, the modified Drucker-Prager yield criterion model with the Drucker-Prager hardening sub-option offered by ABAQUS was utilized in defining the nonlinear plasticity behavior of the confined concrete. The extended Drucker-Prager model can be used in conjunction with the elastic behavior model to define the plasticity of materials by allowing the simultaneous inelastic dilation (increase in volume) [61].

In the Drucker-Prager criterion model, the parameters to be defined include the friction angle of material, flow stress ratio under tri-axial stress, and dilation angle of material. In this study, the friction angle of concrete and flow stress ratio were considered 20° and 0.8, respectively, as proposed by Hu et al. [62]. The determinant parameter that needed to be defined in the Drucker-Prager model was the dilation angle (φ). The dilation angle determines the plasticity flow capacity of the material and confinement effect. Hence, to obtain the accurate ultimate strength of the model, computing an accurate dilation angle is crucial. Eq. (7) was adopted to determine the dilation angle of elliptical-CFRP confined concrete with an inner elliptical steel tube based on the regression analysis as the function of constants $X_{1}$, $X_{2}\text{,}$ and $X_{3}$.

(7) $$\varphi =\left\{ \begin{array}{c} 52.4737\left[{X_{3}}^{-4.8119}-1.6583X_{1}\right]{X_{3}}^{5.2416}\;\\ 51.0685-\left[0.64145\left(X_{1}+{X_{2}}^{-2.8884}\right)\right]{X_{3}}^{0.2828}\; \end{array}\right.$$

where $X_{1}\text{,}$ $X_{2}\text{,}$ and $X_{3}$ were calculated utilizing Eqs. (8)–(10).

(8) $$X_{1}=\frac{E_{frp}t_{frp}}{\left(\sqrt{h^{2}+b^{2}}\right)f_{c}}$$

(9) $$X_{2}=\frac{f_{y}t_{s}}{\left(\sqrt{2{a_{o}}^{2}+2{b_{o}}^{2}}\right)f_{c}}$$

(10) $$X_{3}=\frac{b}{h}$$

where $E_{frp}$, $t_{frp}$, *h*, *b*, ${\;f}_{y}$, $t_{s}$, ${\;2a}_{o}$, ${\;2b}_{o}$, and $f_{c}$ represent the elastic modulus of CFRP (MPa), thickness of CFRP (mm), inner depth of CFRP (mm), inner width of CFRP (mm), yield strength of steel tube (MPa), thickness of steel tube (mm), major axis inner length of steel tube (mm), minor axis inner length of steel tube (mm), and unconfined standard cylinder strength of concrete (MPa), respectively.

#### 2.1.3 Modeling steel tube

**Fig. 3:**
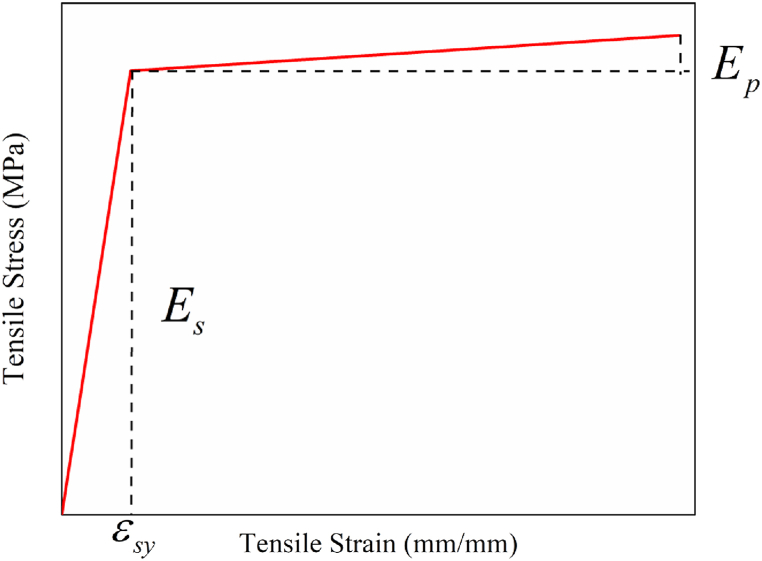
Bi-linear stress-strain curve of inner steel tube.

The behavior of elliptical steel tube defined as bilinear elastic-plastic material with isotropic strain hardening was used to define the material behavior of the inner steel tube. The bilinear elastic-plastic steel material has two regions in the stress-strain curve; the linear elastic region and the plastic region, as depicted in Fig. 3 proposed by Han and Huo [63]. For the linear elastic behavior, the elastic modulus (*E*) and Poisson's ratio (ν) were defined. The plastic behavior was defined using the ultimate strength ($f_{u}$) with the associated plastic strain of the material. It was assumed in the existing literature that the plastic region's modulus made up 1% of the steel's elastic modulus. The tensile stress-strain relation illustrated in Fig. 3 was drawn by using the expression given in Eqs. (11), (12), (13). The key properties of the inner steel tube and end plates employed in FEA are listed in Table 2.

(11) $$\;\sigma_{i}=E_{s}\varepsilon_{s}\;\text{for}\;\varepsilon_{s}\leq \varepsilon_{sy}\;\left(\text{in}\;\text{elastic}\;\text{region}\right)$$

(12) $$\sigma_{i}=f_{sy}+E_{s}\left(\varepsilon_{s}-\varepsilon_{sy}\right)\;\text{for}\;\varepsilon_{s}>\varepsilon_{sy}\;\left(\text{in}\;\text{plastic}\;\text{region}\right)$$

(13) $$\varepsilon_{s}=f_{sy}/E_{s}$$

where $\sigma_{i}$, $E_{s}$, $\varepsilon_{s}$, $f_{y}$, $\varepsilon_{sy}$, $f_{u}$, $\varepsilon_{u}$, and υ are the tensile stress (MPa), elastic modulus (MPa), strain (mm/mm), yield strength (MPa), yield strain (mm/mm), ultimate strength (MPa), ultimate strain (mm/mm), and Poisson's ratio, respectively.

**Table 2:** Key properties of inner steel tube and end plates.

$E_{s}$ (MPa)	$f_{y}$ (MPa)	$\varepsilon_{sy}$	$f_{u}$ (MPa)	$\varepsilon_{u}$	ν	$t_{s}$ (mm)
189,133	785	0.004167	834.7	0.02933	0.267	6
189,133	785	0.004167	834.7	0.02933	0.267	8
191,300	685	0.003581	728.9	0.02295	0.270	6
193,400	782	0.00410	827	0.0254	0.280	3

### 2.2 Finite element type and interaction

#### 2.2.1 Element size and type of CFRP

**Fig. 4:**
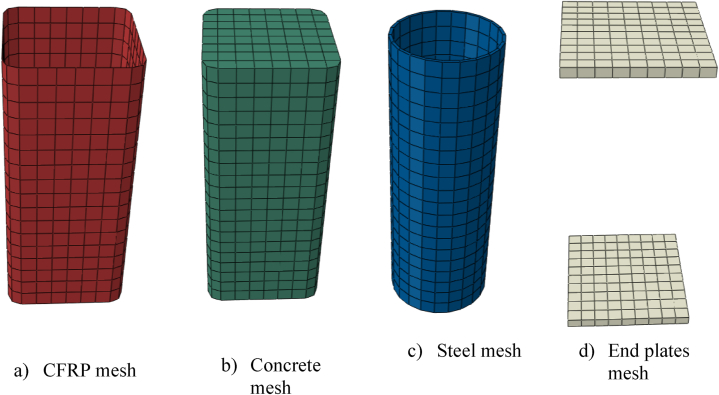
Typical meshed model.

ABAQUS offers finite strain shell elements like S4R with six degrees of freedom at each node. Such shell elements are appropriate for large strain analysis [61]. A linear geometric order shell element with four nodes, S4R, and mesh size of 25 mm were used for meshing the outer rectangular composite CFRP, as displayed in Fig. 4 (a).

#### 2.2.2 Mesh size and type of concrete

A three-dimensional hexahedral 8-node linear solid brick element with reduced numerical integration method, C3D8R, and mesh size of 20 mm were utilized for concrete, as indicated in Fig. 4 (b). C3D8R is a versatile mesh element for different solid body geometry meshing algorithms. Also, such mesh element provides an equivalent solution at low computation time than the triangle and tetrahedral element shapes [61].

#### 2.2.3 Mesh size and type of inner elliptical steel tube

Similar to the concrete mesh element, three-dimensional solid brick with an 8-node hexahedral mesh element shape, C3D8R, was employed for the inner elliptical steel tube and both end plates. However, both end plates were defined as discrete rigid parts, while the inner steel tube was modeled as deformable parts, as presented in Fig. 4 (c) and (d), respectively.

### 2.3 Modeling interaction of object and boundary conditions

**Fig. 5:**
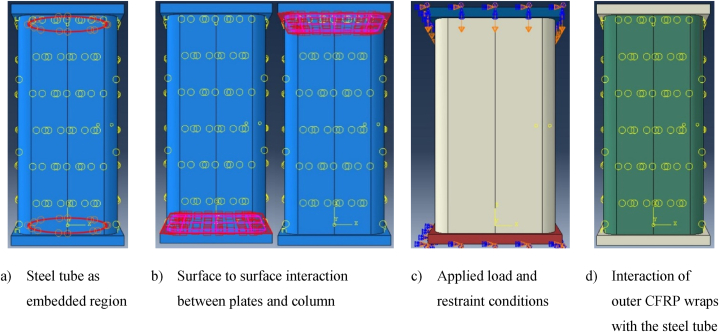
Details of FEM.

CFRP tube, concrete, and steel tube with rigid discrete end plates at each end of the column bases assembled to interact as a single object to form a rectangular solid DSTCC, as demonstrated in Fig. 5 (a)–(d). In this model, the steel tube was embedded in concrete and the external CFRP fused to the concrete surface by surface-to-surface tie constraints. In the tie constraints between CFRP and concrete surface, the contact surface of CFRP was considered as the slave surface while the surface of concrete was defined as the master surface. Also, the two discrete rigid end plates and concrete interact by the surface-to-surface based contact interaction. The surface-to-surface contact interaction is used to describe the contact between deformable body and rigid parts’ surface. When the two bodies are in the surface-to-surface contact, they transfer normal and shear forces across their interaction surface. These forces of the interaction between contacting bodies are defined as friction between the two contacting surfaces. Hence, in this model, the friction between the end plates and the concrete surface was defined by setting the tangential behavior of the contact surfaces using penalty friction formulation and a friction coefficient of 0.6. In addition, to minimize the penetration of the concrete surface into the master end plates, the normal behavior of the surface contact was modeled as hard contact pressure-overclosure. The bottom end plates of the column were fixed, while the top end plates of the column was free to displacement in the direction of the column height, as shown in Fig. 5 (c).

## 3 Validation of FEM

To verify the relevance of the FEA results, 14 different test specimens from two existing related experimental investigations were utilized for comparisons. 12 test specimens were adopted from the experimental research carried out by Ye et al. [10]. In addition, to broaden the verification scope of the proposed model, additional two experimental test specimens were taken from an existing experimental study done by Zeng et al. [1]. The geometric parameters of the test specimens derived from literature [1,10] are outlined in Table 3.

**Table 3:** Specimens data from existing experimental investigations [1,10].

Specimen	FRP tube			Steel tube			Concrete				Reference
	h (mm)	b (mm)	$t_{frp}$ (mm)	${2a}_{s}$ (mm)	${2b}_{s}$ (mm)	$t_{s}$ (mm)	$f_{c}$ (MPa)	*E*_*c* (GPa)_		$\varepsilon_{co}$	
C-1-2	210	210	0.334	180	180	3	32.5	28.3		0.0025	Ye et al. [10]
C-1-4	210	210	0.668	180	180	3					
C-1.5-2	258	172	0.334	220	147	3					
C-1.5-4	258	172	0.668	220	147	3					
C-2-2	298	149	0.334	255	128	3					
C-2-4	298	149	0.668	255	128	3					
CFFT-1-2	210	210	0.334	–	–	–					
CFFT-1-4	210	210	0.668	–	–	–					
CFFT-1.5-2	258	172	0.334	–	–	–					
CFFT-1.5-4	258	172	0.668	–	–	–					
CFFT-2-2	298	149	0.334	–	–	–					
CFFT-2-4	298	149	0.668	–	–	–					
Compressive behavior of double tube concrete columns with an outer square FRP tube (Zeng et al. [1])											
Specimen	w (mm)	$E_{frp}$ (GPa)	$t_{frp}$ (mm)	$d_{s}$ (mm)	$t_{s}$ (mm)	$E_{s}$ (GPa)	$f_{y}$ (MPa)		$f_{c}$ (MPa)	$E_{c}$ (GPa)	$\varepsilon_{co}$
C-260-2	260	239.6	0.668	210	6	191.3	685		37.2	28.9	0.0026
C-260-3	260	239.6	1.002	210	6	191.3	685		37.2	28.9	0.0026

Note: C-1-2 represents DSTCC (double skin tube concrete column with inner elliptical steel tube), and the numbers 1 and 2 stand for the aspect ratio of FRP tube and the number of CFRP layers, respectively. CFFT-1-2 designates concrete-filled CFRP tube without interior steel tube. h is inner depth of FRP tube, b is inner width of FRP tube, $t_{frp}$ is thickness of FRP tube, ${2a}_{s}$ is outer length of major axis of steel tube, ${2b}_{s}$ is outer length of minor axis of steel tube, $t_{s}$ is thickness of steel tube, $f_{c}$ is unconfined cylinder concrete strength, $E_{c}$ is elastic modulus of concrete, $\varepsilon_{co}$ is axial strain of unconfined concrete at ultimate stress, w is clear width of FRP tube, $E_{f}$ is tensile modulus of elasticity of FRP, and $d_{s}$ is outer diameter of steel tube. In a similar way, C-260-2 presents DSTCC with inner steel tube, and the numbers 260 and 2 denote the clear outer width of CFRP and the number of CFRP layers, respectively.

* $\;Difference\;\%=\left(\frac{\mathrm{Exp}-FEA}{\mathrm{Exp}}\times 100\right)$ Exp: experimental test result; FEA: finite element analysis result

**Fig. 6:**
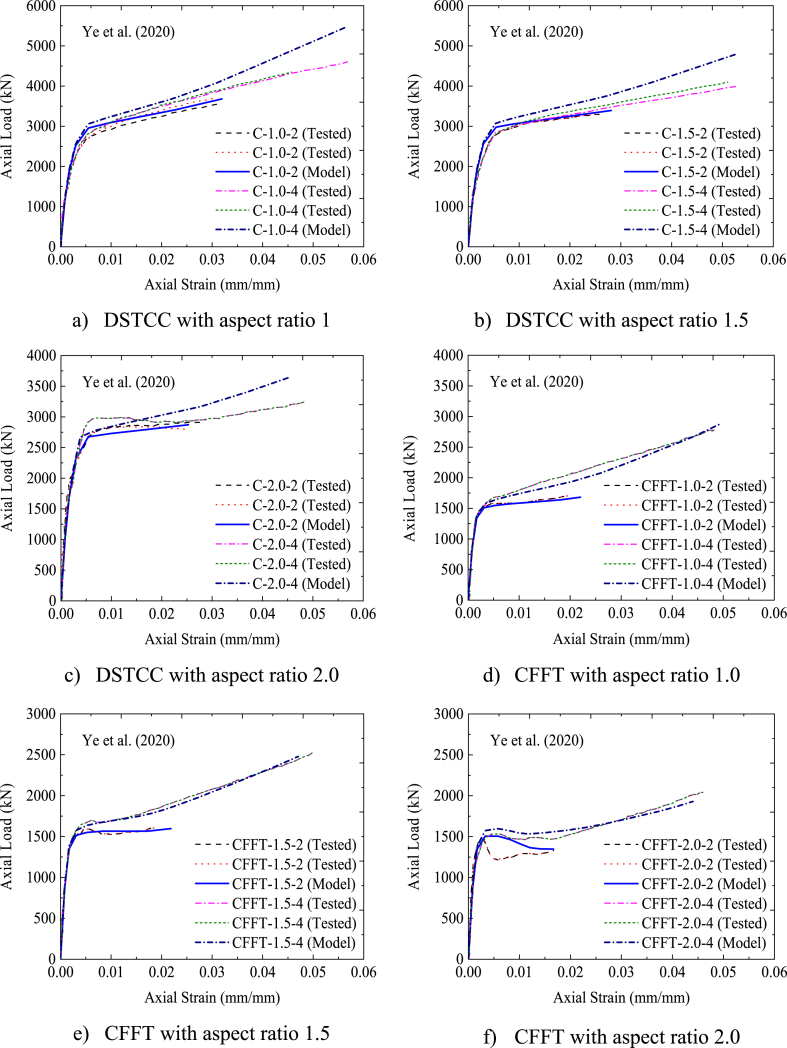

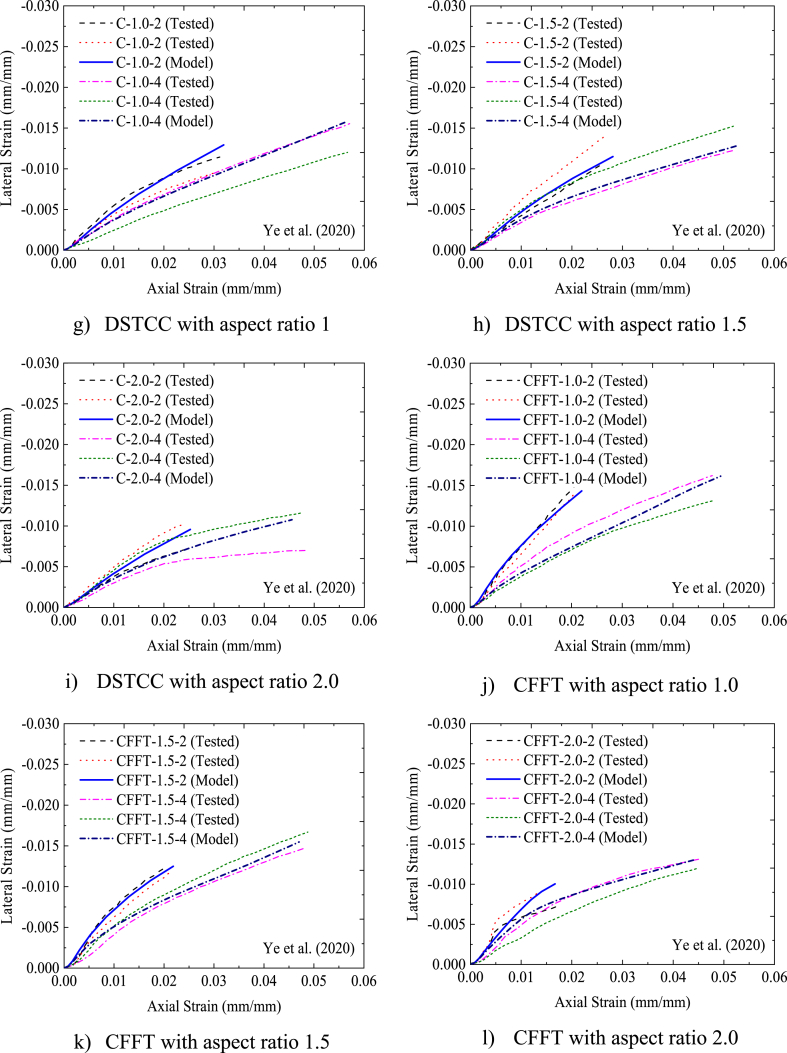
Comparison between tested and modeled axial strain-lateral strain relationships.

**Fig. 7:**
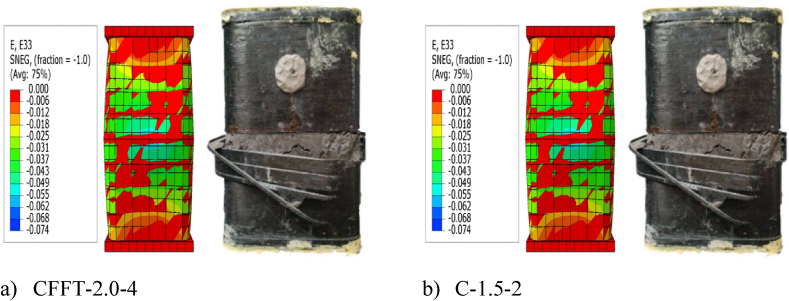
Experimental and FEA failure modes.

The axial load versus axial strain comparisons of FEA and experimental test (Exp) results are indicated in Table 4. As can be seen from Table 4, the ultimate axial load extracted from FEA slightly deviates from the corresponding experimental axial load results by 3.1% on average. While the axial strain and lateral (hoop) strain results from FEA differ from the experimental test results by 2.17% and 13.8% on average, respectively. These slight differences were expected and within tolerable range. The slight deviation of the FEA results from the experimental results may be due to the difference between real and ideal material properties, variation of actual contact interaction between the three materials, and idealized interaction considered in FEMs. The bilinear axial load–axial strain curves obtained from FEA best fit with the axial load–axial strain curves obtained from the experimental test results, as displayed in Fig. 6 (a)–(j). Furthermore, as illustrated in Fig. 7, the deformation model taken from FEA replicates the buckling of the column specimens near the mid-height as observed from the experimental test results. Therefore, FEM implemented in this study is capable of determining the ultimate axial load, axial strain, and lateral strain of rectangular solid DSTCCs.

**Table 4:** Comparison of experimental (Exp) and FEA results.

Specimen	Ultimate axial load ($P_{u}$) (kN)				Axial strain ($\varepsilon_{cu}$) (mm/mm)				Lateral strain ($\varepsilon_{l}$) (mm/mm)			
	Exp	FEA	Exp/FEA	Difference* (%)	Exp	FEA	Exp/FEA	Difference* (%)	Exp	FEA	Exp/FEA	Difference*(%)
C-1-2	3640	3681	0.98	−1.1	0.03120	0.032	0.97	−2.56	−0.01090	−0.0129	0.84	−18.3
C-1-4	4481	5456	0.82	−21.8	0.0510	0.056	0.91	−9.80	−0.01360	−0.0157	0.87	−15.4
C-1.5-2	3628	3393	1.06	6.5	0.02585	0.028	0.92	−8.32	−0.00965	−0.0115	0.84	−19.2
C-1.5-4	4041	4808	0.72	−17.4	0.05190	0.053	0.97	−2.12	−0.01310	−0.0129	1.02	1.5
C-2-2	2877	2870	1.00	0.2	0.02630	0.025	1.05	4.94	−0.00865	−0.0096	0.90	−11.0
C-2-4	3217	3650	0.88	−13.5	0.04535	0.046	0.99	−1.43	−0.00910	−0.0108	0.84	−18.7
CFFT-1-2	1691	1682	1.00	0.5	0.02090	0.022	0.95	−5.26	−0.01475	−0.0143	1.03	3.1
CFFT-1-4	2741	2879	0.95	−5.0	0.04820	0.049	0.98	−1.66	−0.01480	−0.0161	0.92	−8.8
CFFT-1.5-2	1612	1596	1.01	1.0	0.02030	0.022	0.92	−8.37	−0.01090	−0.0125	0.87	−14.7
CFFT-1.5-4	2477	2479	0.99	−0.1	0.04990	0.047	1.06	5.81	−0.01060	−0.0155	0.68	−46.2
CFFT-2-2	1327	1347	0.98	−1.5	0.01680	0.017	0.99	−1.19	−0.00870	−0.0100	0.87	−14.9
CFFT-2-4	2082	1929	1.07	7.3	0.0458	0.044	1.04	3.93	−0.01250	−0.0130	0.96	−4.0
C-260-2	6362	6488	0.98	−1.98	0.0309	0.016	1.93		−0.01172	−0.0081		
C-260-3	6955	6898	1.01	0.81	0.0156	0.0160	0.97		−0.00705	−0.0068		
Difference in average (%)				−3.1				−2.17				**−**13.8

### 3.1 Strength and strain enhancement ratios of confined concrete

In addition to validating the accuracy of the FEA results by comparing them to the experimental test results in terms of the axial load, axial strain, and buckling mode, the strength enhancement ratio *(*$f_{cc}/f_{co})$ and strain enhancement ratio ($\varepsilon_{cu}/\varepsilon_{co})$ of the confined concrete obtained from FEA were also calibrated against the existing experimental test results.

The strength enhancement ratio ($f_{cu}/f_{co})$ indicates that the enhancement of the concrete strength was gained due to the lateral confinement from CFRP and the inner steel tube. The higher strength enhancement ratio implies the effectiveness of confinement provided by CFRP and the inner steel tube. In this study, the confined concrete strength, ${\;f}_{cc}$, was calculated from the axial load carried by concrete per the concrete cross-sectional area. The axial load resisted by concrete was obtained by deducting the ultimate axial load resistance of the inner steel tube from the ultimate axial load achieved from FEA. Eq. (14) was utilized for calculating the ultimate axial resistance of the inner elliptical steel tube.

(14) $$P_{st}=A_{el,st}f_{y}$$

where $P_{st}$ (kN) is the axial load buckling resistance of the inner steel tube, $f_{y}$ (MPa) is the yield strength of the steel tube, and $A_{el,st}$ (mm^2^) is the cross-sectional area of the elliptical steel tube which was determined using Eq. (15).

(15) $$A_{el,st}=P_{m}t_{s}$$

where $t_{s\;}$ (mm) is the thickness of the steel tube and $P_{m}$ (mm) is the mean perimeter of the elliptical steel tube calculated using Eqs. (16), (18), (19), proposed by Ref. [64] as following:

(16) $$P_{m}=\pi \left(a_{m}+b_{m}\right)\left(1+\frac{3h_{m}}{10+\sqrt{4-3h_{m}}}\right)$$

in which,

(17) $$a_{m}=\frac{2a_{o}-t}{2}$$

(18) $$b_{m}=\frac{2b_{o}-t}{2}$$

(19) $$h_{m}=\frac{{\left(a_{m}-b_{m}\right)}^{2}}{{\left(a_{m}+b_{m}\right)}^{2}}$$

Also, the nominal cross-sectional area of concrete ($A_{nc}$) in mm^2^ was determined using Eq. (20).

(20) $$A_{nc}=hb-\left(4-\pi \right){r_{c}}^{2}$$

where ${2a}_{o}$ (mm) and ${2b}_{o}$ (mm) are the major and minor axes outer dimensions of the inner elliptical steel tube, respectively, and $r_{c}$ (mm) is the radius of the section corner.

The comparisons of the strength enhancement $\left(f_{cc}/f_{co}\right)$ and strain enhancement ($\varepsilon_{cu}/\varepsilon_{co})$ ratios from FEA and the existing experimental tests [10] are listed in Table 5. As can be seen from Table 5, both the strength enhancement and strain enhancement ratios determined by FEA demonstrate reliable agreement against the experimental test results with minor discrepancies. Hence, the proposed FEM in this study is capable of reproducing the experimental test results in investigating the performance and behavior of CFRP-confined concrete columns with inner elliptical high-strength steel tube.

**Table 5:** Confined concrete strength and strain enhancement ratios.

Specimen	$f_{cc}$ (MPa)	$f_{cc}$ (MPa)	Strength enhancement ratio ($f_{cc}/f_{co}$)		Strain enhancement ratio ($\varepsilon_{cu}/\varepsilon_{co}$)	
	Exp	FEA	Exp	FEA	Exp	FEA
C-1.0-2	58.6	54.92	1.80	1.69	12.45	12.77
C-1.0-4	79.3	95.88	2.44	2.95	20.55	22.51
C-1.5-2	49.6	46.62	1.53	1.43	10.35	11.25
C-1.5-4	67.75	79.07	2.08	2.43	20.8	21.21
C-2.0-2	39.35	33.06	1.22	1.02	10.5	10.11
C-2.0-4	47.55	50.93	1.46	1.57	18.1	18.22
CFFT-1.0-2	39	38.82	1.20	1.19	8.4	8.36
CFFT-1.0-4	63	66.45	1.95	2.04	19.3	19.28
CFFT-1.5-2	37	36.62	1.14	1.13	8.1	8.12
CFFT-1.5-4	56.8	56.86	1.75	1.75	20.0	19.96
CFFT-2.0-2	30.4	30.88	0.94	0.95	6.7	6.72
CFFT-2.0-4	47.7	38.82	1.47	1.36	18.3	18.32

## 4 Parametric study

The validated FEM presented in the previous section was used for the extensive parametric study. The effects of the concrete strength, inner steel tube thickness, size of inner steel tube, and CFRP thickness on the strength and deformation response of rectangular DSTCCs are discussed in this section. For all variables of the parametric study, test models with four different aspect ratios wrapped by 2 and 4 layers of CFRP were designed, as given in Table 6. For examining the effect of the concrete strength on the rectangular DSTCC specimens, different grades of the concrete strength were considered. To figure out the effect of the inner elliptical steel tube thickness, test specimens with the steel tube thicknesses of 6 mm and 8 mm were designed. Moreover, to comprehend the impact of the cross-sectional size of the inner steel tube, test specimens with two different inner steel tube sizes were designed (Table 6). In addition, to evaluate the confinement effectiveness of the inner steel tube, the strength and response of the DSTCC test specimens were compared to those of the rectangular CFRP-confined concrete columns without inner steel tubes (CFFTs). All the test specimens’ heights and corner radii of the sections were 450 mm and 30 mm, respectively.

**Table 6:** Geometric details of parametric study of test specimens.

Specimen	CFRP			Elliptical high-strength steel tube					
	h (mm)	b (mm)	$t_{frp}$ (mm)	${2a}_{o}$ (mm)	${2b}_{o}$ (mm)	${2a}_{i}$ (mm)	${2b}_{i}$ (mm)	$t_{s}$ (mm)	$A_{s}$ (mm^2^)
C-1.0-2-C60-T8	210	210	0.334	180	164	164	164	8	17298.29
C-1.0-4-C60-T8	210	210	0.668	180	164	164	164	8	17298.29
C-1.5-2-C60-T8	258	172	0.334	220	204	204	131	8	17650.29
C-1.5-4-C60-T8	258	172	0.668	220	204	204	131	8	17650.29
C-2.0-2-C60-T8	298	149	0.334	255	239	239	112	8	18454.86
C-2.0-4-C60-T8	298	149	0.668	255	239	239	112	8	18454.86
C-2.5-2-C60-T8	340	136	0.334	290	274	274	100	8	19611.43
C-2.5-4-C60-T8	340	136	0.668	290	274	274	100	8	19611.43
C-1.0-2-C30-T8	210	210	0.334	180	164	164	164	8	17298.29
C-1.0-4-C30-T8	210	210	0.668	180	164	164	164	8	17298.29
C-1.5-2-C30-T8	258	172	0.334	220	204	204	131	8	17650.29
C-1.5-4-C30-T8	258	172	0.668	220	204	204	131	8	17650.29
C1.0-2-C60-T6	210	210	0.334	180	180	168	168	6	13124.57
C-1.0-4-C60-T6	210	210	0.668	180	180	168	168	6	13124.57
C-1.5-2-C60-T6	258	172	0.334	220	147	208	135	6	13388.57
C-1.5-4-C60-T6	258	172	0.668	220	147	208	135	6	13388.57
C-2.0-2-C60-T6	298	149	0.334	255	128	243	116	6	13992.00
C-2.0-4-C60-T6	298	149	0.668	255	128	243	116	6	13992.00
C-2.5-2-C60-T6	340	136	0.334	290	116	278	104	6	14859.43
C-2.5-4-C60-T6	340	136	0.668	290	116	278	104	6	14859.43
C-1.0-2-50	210	210	0.334	90	90	84	84	3	3281.14
C-1.0-4-50	210	210	0.668	90	90	84	84	3	3281.14
C-1.5-2-50	258	172	0.334	110	74	104	68	3	3347.14
C-1.5-4-50	258	172	0.668	110	74	104	68	3	3347.14
C-2.0-2-50	298	149	0.334	128	64	122	58	3	3498.00
C-2.0-4-50	298	149	0.668	128	64	122	58	3	3498.00
C-2.5-2-50	340	136	0.334	145	58	139	52	3	3714.86
C-2.5-4-50	340	136	0.668	145	58	139	52	3	3714.86
C-1.0-2-C60-T6-50	210	210	0.334	90	90	78	78	6	6336.00
C-1.0-4-C60-T6-50	210	210	0.668	90	90	78	78	6	6336.00
C-1.5-2-C60-T6-50	258	172	0.334	110	74	98	62	6	6468.00
C-1.5-4-C60-T6-50	258	172	0.668	110	74	98	62	6	6468.00
C-2.0-2-C60-T6-50	298	149	0.334	128	64	116	52	6	6769.71
C-2.0-4-C60-T6-50	298	149	0.668	128	64	116	52	6	6769.71
C-2.5-2-C60-T6-50	340	136	0.334	145	58	133	46	6	7203.43
C-2.5-4-C60-T6-50	340	136	0.668	145	58	133	46	6	7203.43
CFFT-1.0-2-C60	210	210	0.334	–	–	–	–	–	–
CFFT-1.0-4-C60	210	210	0.668	–	–	–	–	–	–
CFFT-1.5-2-C60	258	172	0.334	–	–	–	–	–	–
CFFT-1.5-4-C60	258	172	0.668	–	–	–	–	–	–
CFFT-2.0-2-C60	298	149	0.334	–	–	–	–	–	–
CFFT-2.0-4-C60	298	149	0.668	–	–	–	–	–	–

## 5 Results and discussion of FEA

### 5.1 Effect of concrete strength grade

**Fig. 8:**
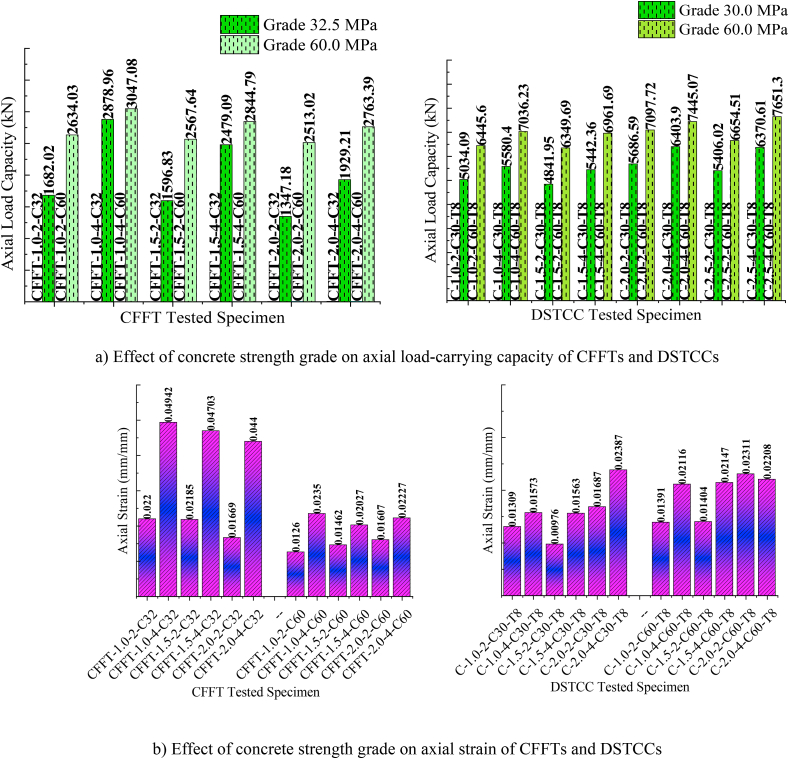
Effect of concrete strength grade on axial load-carrying capacity and axial strain of CFFTs and DSTCCs.

To investigate the effect of the concrete strength, the unconfined cylinder concrete strengths of 30 MPa, 32.5 MPa, and 60 MPa were used in the test models with various aspect ratios and CFRP layers. Fig. 8 shows the effect of the concrete strength on the ultimate axial load-carrying capacity and ultimate axial strain of the DSTCC and CFFT columns. The ultimate axial load-carrying capacity of the DSTCC columns enhanced as the strength of concrete increased (Fig. 8 (a)). As can be seen from Fig. 8 (b), the ultimate axial strain of the CFFT columns decreased as the concrete strength increased, similar existing studies also approve this finding [16,27]. However, an improvement was noticed in the strain of the DTCC columns with higher concrete strength, which is the result of better confinement by the inner steel tube.

**Fig. 9:**
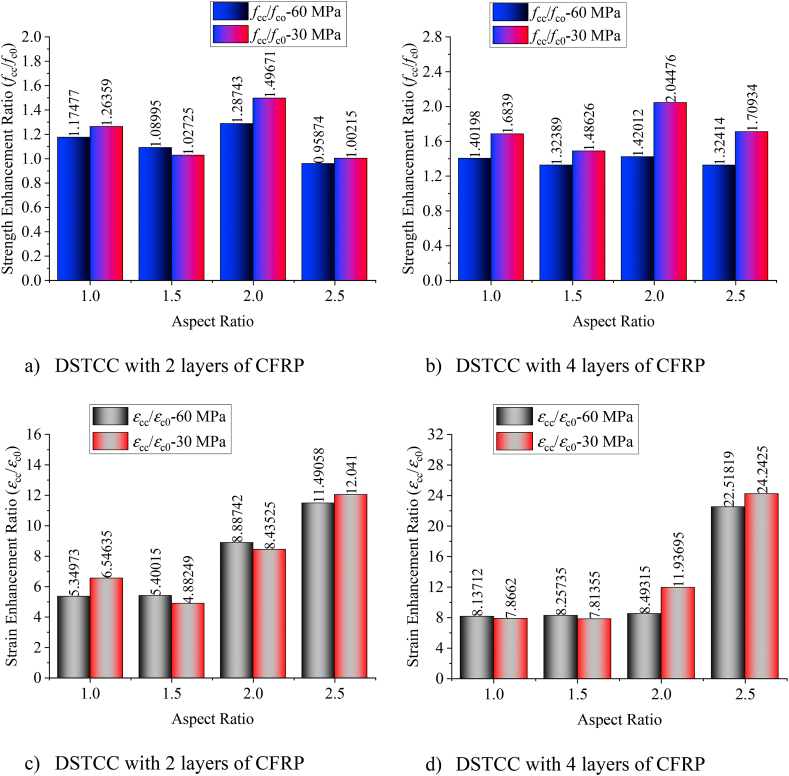
Effect of concrete strength grade on strength and strain enhancement ratios of DSTCCs.

However, the enhancement strength ratio ($f_{cc}/f_{co})$ of the models with the unconfined concrete strength of 30 MPa demonstrated a remarkably larger strength enhancement ratio than the test specimens with 60 MPa concrete strength (Table 7). The ratio of the strength enhancement ($f_{cc}/f_{co})$ implies the concrete strength gained from confinement in proportion to its unconfined concrete strength. The larger enhancement ratio points out the effectiveness of confinement from CFRP and the inner steel tube. Fig. 9 illustrates the effect of the concrete strength grade on the strength and strain enhancement ratios. As can be seen from Fig. 9 (a) and (b), the test specimens with 30 MPa concrete strength revealed a better strength enhancement ratio than the test specimens with 60 MPa, except for the test specimens confined by 2 layers of CFRP and aspect ratio of 1.5. In line with this finding, Ozbakkaloglu and Oehlers [26] reported that the confined concrete strength ratio ($f_{cc}/f_{co})$ decreased with increasing the concrete strength. Also, with increasing the CFRP confining thickness, the low-strength concrete exhibited a larger strength enhancement ratio (Fig. 9 (b)).

**Table 7:** Effect of concrete strength.

Specimen	$P_{u}$ (kN)	$f_{cc}$ (MPa)	$\varepsilon_{cu}$ (mm)	$\varepsilon_{l}$	($f_{cc}/f_{co}$)	($\varepsilon_{cu}/\varepsilon_{co}$)
C-1.0-2-C60-T8	6445.6	70.49	0.0139	−0.0110	1.17	5.35
C-1.0-4-C60-T8	7036.2	84.12	0.0212	−0.0132	1.40	8.14
C-1.5-2-C60-T8	6349.6	65.40	0.0140	−0.0117	1.09	5.40
C-1.5-4-C60-T8	6961.6	79.43	0.0215	−0.0138	1.32	8.26
C-2.0-2-C60-T8	7097.7	77.25	0.0231	−0.0144	1.29	8.89
C-2.0-4-C60-T8	7445.0	85.21	0.0221	−0.0113	1.42	8.49
C-2.5-2-C60-T8	6654.5	57.52	0.0299	−0.0129	0.96	11.49
C-2.5-4-C60-T8	7651.3	79.45	0.0585	−0.0111	1.32	22.52
C-1.0-2-C30-T8	5034.0	37.91	0.0131	−0.0129	1.26	6.55
C-1.0-4-C30-T8	5580.4	50.52	0.0157	−0.0104	1.68	7.87
C-1.5-2-C30-T8	4841.9	30.82	0.0098	−0.0117	1.03	4.88
C-1.5-4-C30-T8	5442.3	44.59	0.0156	−0.0124	1.49	7.81
C-2.0-2-C30-T8	5686.5	44.90	0.0169	−0.0116	1.50	8.44
C-2.0-4-C30-T8	6403.9	61.34	0.0239	−0.0118	2.04	11.94
C-2.5-2-C30-T8	5406.0	30.06	0.0241	−0.0114	1.00	12.04
C-2.5-4-C30-T8	6370.6	51.28	0.0485	−0.0110	1.71	24.24

Note: $P_{u}$ is ultimate axial load, $f_{cc}$ is confined concrete strength, $\varepsilon_{cu}$ is ultimate axial strain, $\varepsilon_{co\;}$ is unconfined concrete strain at ultimate axial load, $\varepsilon_{l\;}$ is lateral strain at CFRP rupture, $f_{cc}/f_{co}$ is enhancement ratio of confined concrete strength, and $\varepsilon_{cu}/\varepsilon_{co}$ is strain enhancement ratio.

The effect of the concrete strength on the strain enhancement ratio $\left(\varepsilon_{cu}/\varepsilon_{co}\right)$ of each test specimen is displayed in Table 7. The strain enhancement ratio indicates the deformability behavior achieved by confining concrete between CFRP and the inner steel tube. Fig. 9 (c) and (d) shows the impact of the concrete grade along with different thicknesses of CFRP on the strain enhancement ratio with different aspect ratios of DSTCCs. As can be observed from Fig. 9 (d), the test specimens with 30 MPa concrete strength confined by 4 layers of CFRP achieved a larger strain enhancement ratio than the models with 60 MPa concrete strength. However, slight difference in the strain enhancement ratio $\left(\varepsilon_{cu}/\varepsilon_{co}\right)$ was witnessed between the concrete strengths of 30 MPa and 60 MPa confined by 2 layers of CFRP. Generally, relative to the unconfined ultimate strain, lower confined concrete strength in DSTCCs provided large deformation before the tensile rupture of CFRP. This behavior of DSTCCs is not only limited to the concrete compressive strength, but also the thickness of the confining CFRP and aspect ratio have a significant influence.

### 5.2 Effect of inner steel tube thickness

Test specimens with 6 mm and 8 mm thicknesses of the inner steel tube were modeled to examine the effect of the inner steel tube thickness on the capacity and response of DSTCCs. In addition, identical test specimens without the inner steel tube were compared to the DSTCC specimens to comprehend the effectiveness of the inner steel tube in DSTCCs.

**Fig. 10:**
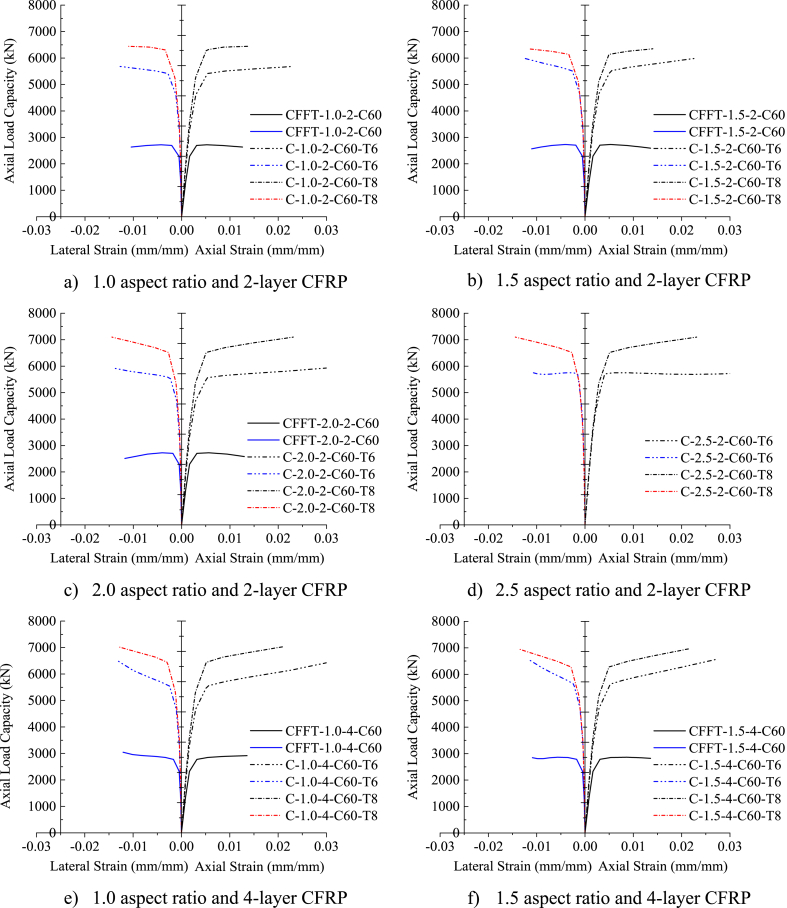

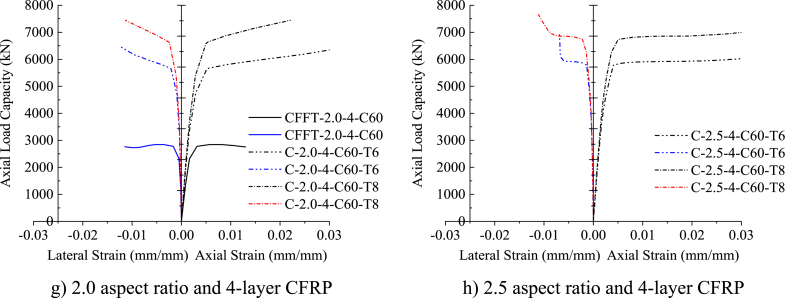
Effect of inner steel tube thickness.

Fig. 10 (a)–(h) depicts the axial load versus axial and lateral strain responses of DSTCCs with different thicknesses of the inner elliptical steel tube. As can be seen from the figure, all the test specimens represent a bilinear curve with a monotonic ascending second curve before attaining the CFRP tensile rupture at the ultimate load. This demonstrates that the thickness of the inner steel tube had no noticeable impact on the axial stress-strain responses. However, for the large aspect ratios of 2.0 and 2.5, the second ascending curve tended to be horizontal (Fig. 10 (d), (g), (h)). Saenz and Pantelides [40] reported a similar response of the axial load-axial strain curves of DSTCCs under axial compression load. The curves displayed in Fig. 10 (a)–(h) also illustrate the effect of the inclusion of an elliptical steel tube in DSTCCs. As can be seen from Fig. 10 (a)–(h), the specimens with the inner steel tube (DSTCCs) ruptured at higher axial load and ultimate strain than the specimens without the inner steel tube (CFFTs) of the same aspect ratio and thickness of CFRP.

The ultimate axial load, ultimate strain at CFRP tensile rupture, confined concrete strength, and strain enhancement ratio of DSTCCs with 6 mm and 8 mm thicknesses of the inner elliptical steel tube are summarized in Table 8. Increasing the inner steel tube thickness in DSTCCs from 6 mm to 8 mm without altering the tube aspect ratio remarkably enhanced the axial load-carrying capacity of the columns. For instance, for the model C1.0-2-C60, increasing the inner steel tube thickness from 6 mm to 8 mm improved the axial load-carrying capacity by 13.4%. Previous relevant research done by Lu et al. [65] indicated that the axial load-carrying capacity of such columns enhanced with the increase of the inner steel tube thickness. Also, İpek et al. [66] demonstrated that increasing the thickness of the outer steel tube enhanced the axial load-carrying capacity of the columns.

**Table 8:** Effect of inner steel tube thickness.

Specimen	$P_{u}$ (kN)			$\varepsilon_{cu}$ (mm/mm)		$\varepsilon_{l}$ (mm/mm)		$f_{cc}/f_{co}$			$\varepsilon_{cu}/\varepsilon_{co}$		
	T = 6 mm	T = 8 mm	Difference (%)	T = 6 mm	T = 8 mm	T = 6 mm	T = 8 mm	T = 6 mm	T = 8 mm	Difference (%)	T = 6 mm	T = 8 mm	Difference (%)
C1.0-2-C60	5683.13	6445.6	13.4	0.0231	0.0139	−0.0127	−0.0110	1.20	1.17	−1.8	8.9	5.3	−39.8
C-1.0-4-C60	6484.55	7036.23	8.5	0.0315	0.0212	−0.0130	−0.0132	1.50	1.40	−6.8	12.1	8.1	−32.9
C-1.5-2-C60	5996.34	6349.69	5.9	0.0231	0.0140	−0.0127	−0.0117	1.28	1.09	−14.7	8.9	5.4	−39.3
C-1.5-4-C60	6552.63	6961.69	6.2	0.0269	0.0215	−0.0116	−0.0138	1.49	1.32	−11.2	10.4	8.3	−20.3
C-2.0-2-C60	5934.57	7097.72	19.6	0.0304	0.0231	−0.0144	−0.0144	1.19	1.29	8.4	11.7	8.9	−23.9
C-2.0-4-C60	6471.43	7445.07	15.0	0.0342	0.0221	−0.0124	−0.0113	1.39	1.42	1.9	13.2	8.5	−35.4
C-2.5-2-C60	5776.92	6654.51	15.2	0.0373	0.0299	−0.0112	−0.0129	1.00	0.96	−3.8	14.4	11.5	−19.9
C-2.5-4-C60	6927.66	7651.3	10.4	0.0689	0.0585	−0.0069	−0.0111	1.42	1.32	−6.7	26.5	22.5	−15.1

Note: Negative sign indicates reduction of effect by increasing thickness of inner elliptical steel tube from 6 mm to 8 mm.

It can be seen from Table 8 that the enhancement ratio of the confined strength achieved by the DSTCC specimens with the inner steel tube thickness of 6 mm was larger than the specimens with the inner steel tube thickness of 8 mm for the same aspect ratio and thickness of CFRP except for the aspect ratio of 2.0.

This result implies that even though increasing the inner steel tube thickness boosted the axial load-carrying capacity of DSTCCs, in terms of the confined concrete strength enhancement, the thinner steel tube showed better effectiveness in the concrete confinement. A similar finding is observed for the strain enhancement ratio in Fig. 10 (a)–(h). The DSTCC specimen with a 6 mm thick inner steel tube exhibited larger deformation than the specimen with an 8 mm thick inner steel tube. From the numerical results given in Table 8, it can be seen that increasing the thickness of the inner steel tube from 6 mm to 8 mm for the specimen C1.0-2-C60 reduced the strain enhancement ratio by 39.8%. In agreement with this result, the research by Ref. [42] ensured this finding.

$$Difference\;\%=\left(\frac{Specimen\;with\;T8\;-\;Specimen\;with\;T6}{Specimen\;with\;T8}\right)\times 100$$

### 5.3 Effect of cross-sectional size of inner steel tube

**Fig. 11:**
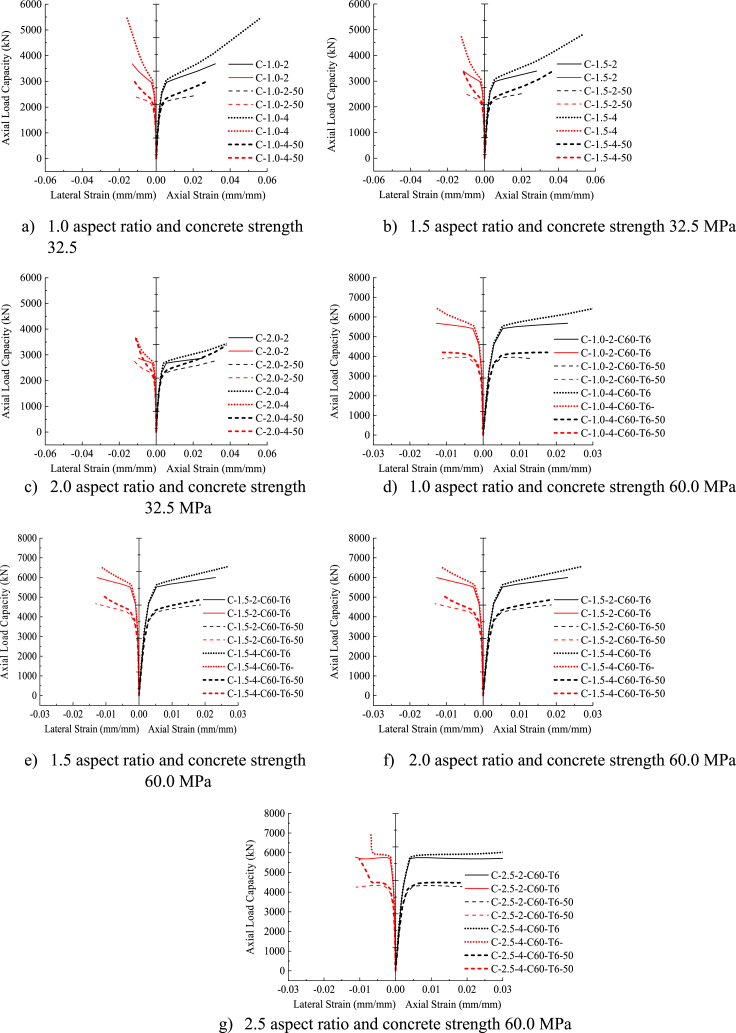
Effect of cross-sectional size of inner steel tube.

To evaluate the effect of the cross-sectional size of the inner steel tube on the response of DSTCCs under an axial compression load, identical test specimens with different inner steel tube cross-sectional sizes were used. Table 9 depicts the ultimate axial load, ultimate axial strain, lateral strain, and confined concrete strength and strain enhancement ratios of the specimens. The specimens labeled with 50 after the thickness of the inner steel tube (for example C-1.0-2-C60-T6-50) signify that the inner steel tube cross-sectional sizes in both major and minor axes reduced by half (50%) of the original specimens (C-1.0-2-C60-T6). Fig. 11 (a)–(g) illustrates the effect of the inner steel tube cross-sectional size on the response of the specimens with different aspect ratios. The figure points out that the cross-sectional sizes of the inner steel tube had no significant impact on the axial load and strain curves of the specimens. The specimens with the reduced inner steel tube cross-sectional sizes provided similar bilinear axial load-axial strain curves and similar axial load-lateral strain curves to those of the original specimens of the same aspect ratios and CFRP thicknesses.

**Table 9:** Key analysis results on effect of cross-sectional size of inner steel tube.

Specimen	Original	Half (50%)	Original	Half (50%)	Original	Half (50%)	Original	Half (50%)	Original	Half (50%)
	$P_{u}$ (kN)		$\varepsilon_{cu}$ (mm/mm)		$\varepsilon_{l}$ (mm/mm)		$f_{cc}/f_{co}$		$\varepsilon_{cu}/\varepsilon_{co}$	
C1.0-2-C60- T6	5683.13	3892.73	0.0231	0.0127	−0.0127	−0.0111	1.20	1.02	8.9	4.9
C-1.0-4-C60-T6	6484.55	4202.12	0.0315	0.0176	−0.0130	−0.0115	1.50	1.14	12.1	6.8
C-1.5-2-C60-T6	5996.34	4668.09	0.0231	0.0216	−0.0127	−0.0131	1.28	1.29	8.9	8.3
C-1.5-4-C60-T6	6552.63	5102.63	0.0269	0.0256	−0.0116	−0.0111	1.49	1.46	10.4	9.9
C-2.0-2-C60-T6	5934.57	4522.28	0.0304	0.0221	−0.0144	−0.0126	1.19	1.20	11.7	8.5
C-2.0-4-C60-T6	6471.43	4896.9	0.0342	0.0267	−0.0124	−0.0105	1.39	1.35	13.2	10.4
C-2.5-2-C60-T6	5776.92	4253.52	0.0373	0.0239	−0.0112	−0.0110	1.00	1.01	14.4	9.3
C-2.5-4-C60-T6	6927.66	5663.97	0.0689	0.0719	−0.0069	−0.0101	1.42	1.53	26.5	27.9
C-1.0-2-C32.5-T3	3681.61	2435.18	0.0319	0.0201	−0.0129	−0.0126	1.69	1.27	12.8	8.0
C-1.0-4-C32.5-T3	5456.15	3023.04	0.0563	0.0280	−0.0157	−0.0120	2.95	1.69	22.5	11.2
C-1.5-2-C32.5-T3	3393.31	2530.42	0.0281	0.0215	−0.0115	−0.0106	1.43	1.31	11.2	8.6
C-1.5-4-C32.5-T3	4808.19	3439.94	0.0530	0.0379	−0.0129	−0.0119	2.43	1.96	21.2	15.2
C-2.0-2-C32.5-T3	2870.568	2757.31	0.0253	0.0314	−0.0096	−0.0118	1.02	1.45	10.1	12.6
C-2.0-4-C32.5-T3	3650.427	3762.7	0.0456	0.0471	−0.0108	−0.0117	1.57	2.16	18.2	18.8

Half (50%) indicates reduction of inner steel tube size by half (50%) in both major and minor axes.

However, as depicted in Fig. 11 (a)–(g) and also the analysis results listed in Table 9, the inner steel tube had a significant impact on the structural behavior of DSTCCs. Specimens with larger inner steel tube cross-sectional sizes failed at high ultimate axial load and underwent larger deformation before CFRP ruptured than the corresponding specimens with the reduced inner steel tube cross-sectional sizes. The interesting finding observed in this study is that the confined concrete strength enhancement ratio ($f_{cc}/f_{co}$) difference between the specimens with the reduced inner steel tube cross-sectional sizes and original specimens with larger inner steel tube cross-sectional sizes reduced as the aspect ratio of the specimens increased. Surprisingly, the analysis results revealed that the confined concrete strength of the specimens with large aspect ratios of 2.0 and 2.5and reduced inner steel tube cross-sectional sizes enhanced slightly better than or equal to their corresponding specimens with large inner steel tube cross-sectional sizes, as presented in Table 9. Therefore, for a large aspect ratio, the confinement effect of a small inner steel tube cross-sectional size was more effective than a large one.

## 6 Data processing and analysis

**Fig. 12:**
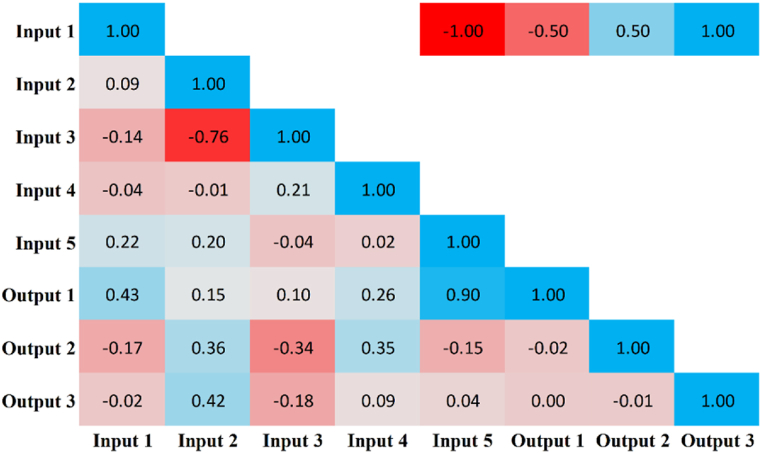
Pearson correlation with heat map.

The current study developed multiple ML models to estimate the confined axial load-carrying capacity, confined axial strain, and confined lateral strain using a final dataset of 76 data points with five input features. The concrete strength (MPa), section width (mm), section depth (mm), total number of CFRP multiplied by the FRP elastic modulus, and the area of the inner steel tube multiplied by its yield strength were all taken into account as input parameters for the created models. These variables were designated as input 1, input 2, input 3, input 4, and input 5, respectively. The confined axial load-carrying capacity, confined axial strain, and confined lateral strain were considered as output variables to develop the models and were respectively denoted as output 1, output 2, and output 3. Input and output parameters are displayed with descriptive statistics in Table 10, which indicates the diverse range of the dataset. Following the descriptive study, a statistical analysis was also performed to establish the degree of correlation between the aforementioned variables using the Pearson correlation (Fig. 12). It is clear from Fig. 12 that output 1 is highly correlated with input 5. Also, it can be seen that for output 1, all the input variables have a positive correlation. For output 2, all five input variables demonstrate significant correlation, with input 1, input 3, and input 5 showing negative correlation, whereas input 2 and input 4 depict positive correlation. For output 3, there are positive and negative correlations with input 2 and input 3, respectively, whereas input 1, input 4, and input 5 were found to have very little correlation.

**Table 10:** Descriptive statistics of input and output parameters.

Parameter	Maximum	Minimum	Std. Dev	Variance	Mean	Median	Std. Error	Skewness	Kurtosis
Input 1	60	30	13.53	183.14	43.46	32.5	1.552	0.40	−1.841
Input 2	340	210	43.11	1858.69	266.84	258	4.945	0.142	−0.973
Input 3	260	136	35.77	1280.02	178.15	172	4.103	0.802	−0.163
Input 4	240.07	78.45	43.44	1887.40	121.55	156.91	4.983	0.353	−0.782
Input 5	3848.74	0	1327.75	1762942	1634.82	1338.21	152.30	0.288	−1.297
Output 1	7651.3	1327	1778.72	3163872	4389.71	4367.51	204.03	0.043	−1.275
Output 2	0.0826	0.0097	0.0154	0.0002	0.0301	0.0239	0.0017	1.234	1.156
Output 3	−0.0047	−0.0161	0.0019	0	−0.0116	−0.0116	0.0002	0.610	1.952

## 7 ML models

### 7.1 Extreme gradient boosting

**Fig. 13:**
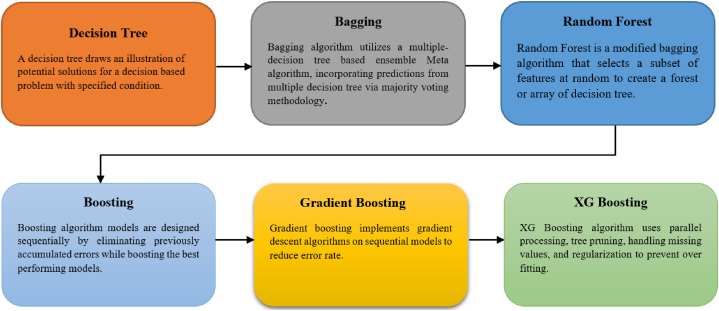
Evolution of decision tree algorithms to XG boosting.

Extreme gradient boosting (XG boosting or XGB) is a highly scalable gradient-based decision tree ensemble [67,68]. It is an improvement over Friedman's gradient boosting method [67,69,70]. Numerous innovative applications have sprung from the algorithm, which also served as the inspiration for many of more contemporary developments. The XG boosting algorithms are, in general, the refined form of older decision tree methods. The evolution of decision tree algorithms to XG boosting is illustrated in Fig. 13.

One of the powerful tools for creating ensemble models was bagging, which was first developed by Breiman [71]. It is the process of training many base learners on a dataset and then selecting the base learners' output as the final outcome. Each iteration of the bagging algorithm's training process involves creating a bootstrap replicate of the preceding iteration's training process [72,73]. This represents a training phase with n cases, followed by a training phase T′ formed by consistently creating *n* examples from training phase T. In the training phase, there is a probability 1−[1−1n]n that out of every *n* times cases are selected at random, at least one will be designated [74]. On the other hand, boosting is a sequential ensemble approach. Weaker predictor models are combined to increase predicted accuracy using this strategy. Models are generated after each iteration's weighting procedure. Boosting concentrates on fresh learning on data with a low accuracy value produced in a previous iteration employing a sequential training approach. To maximize the objective function, XG boosting minimizes a loss function, much like gradient boosting does. Eq. (21) indicates the utilization of a loss function variant to control the complexity of a tree. In Eq. (22), *T* designates the total number of leaves within the tree, while *w* represents the output scores assigned to each leaf [75].

(21) $$L_{XGB}=\sum_{i=1}^{N}L\left(y_{i},F\left(x_{i}\right)\right)+\sum_{m=1}^{M}\Omega \left(h_{m}\right)$$

(22) $$\Omega \left(h\right)=\gamma T+\frac{1}{2}⋋{\left\|w\right\|}^{2}$$

**Fig. 14:**
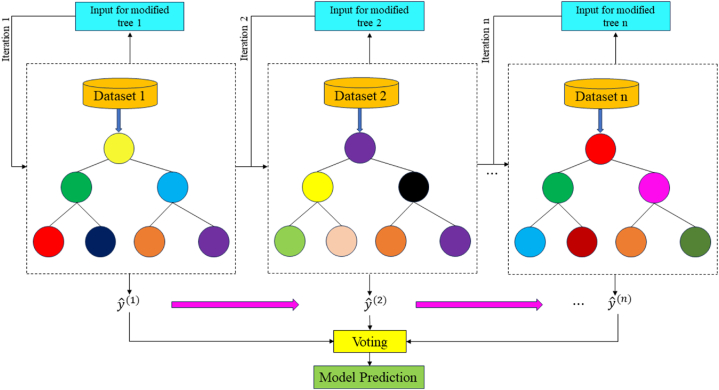
Architecture of XGB [75].

Incorporating this loss function into the split criterion of decision trees yields a pre-pruning approach. When γ increases, the complexity of a tree decreases. The parameter γ determines the threshold of loss reduction gain required to perform an internal node split. In XGB, shrinkage is a regularization parameter that reduces the step size of the additive expansion. The intricacy of the trees can be reduced by the employment of various other methods such as the depth of the trees, etc. The regression process of XGB is displayed in Fig. 14.

### 7.2 Random forest

**Fig. 15:**
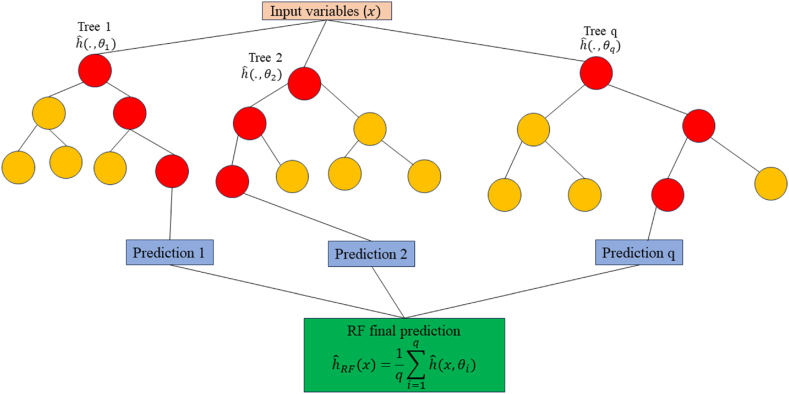
Architecture of RF model [75].

A more advanced version of bagging is the random forest (RF) method. It has been widely utilized in recent years to predict the strength of various types of concrete because of its superior data classification ability [[76], [77], [78]]. In addition, RF extends the training of the decision tree by randomly selecting attributes on the basis of the decision tree's integration as the basic classifier. By measuring the impurity of the feature division result and computing the information gain, it chooses the splitting feature. The weighted average of the results from each decision tree, separated from the root node in line with the feature division requirement and the principle of minimum purity of nodes until the rule is satisfied, constitutes the final prediction result. When training a single decision tree within RF, the RF algorithm randomly chooses subsets from the original dataset with replacement [84]. It then randomly selects features and determines the best features among them to split the tree nodes. This prevents the RF model from overlearning the training set's features and lowers the variance of the model. The construction of each tree by RF using a replacement sample is shown in Fig. 15. Fig. 15 and Eq. (23) depict how the RF model's prediction function $\left({\hat{h}}_{RF}\right)$ may be represented as the mean of each CART's predictions, where $\hat{h}$ *(*$x,\theta_{i})$ denotes the model's prediction of the target and $\theta_{i}$ is the bootstrapped sample used to train the *i*CART model [75].

(23) $${\hat{h}}_{RF}\left(x\right)=\frac{1}{q}\sum_{i=1}^{q}\hat{h}\left(x,\theta_{i}\right)$$

### 7.3 Artificial neural network

Artificial neural networks (ANNs), which were inspired by biological neural networks, are intelligence tools that can easily learn patterns and predict outcomes in high-dimensional space [79,80]. They are able to map inputs to outputs in a dataset despite the complexity and noise. The typical architecture of an ANN includes an output layer, hidden layers, and an input layer [81,82]. The input layer sends the value of inputs to the hidden layer's accessible neurons. An activation function transforms the weighted sum of inputs calculated inside each neuron along with a bias value. The neurons in the following layer get the output signal at the end. Eq. (24), in which $x_{i}$ and $y_{j}$ represent the nodal values in layer *i*'s previous and layer *j*'s current, respectively, provides an explanation of the mathematical procedure. From the previous layer, a total of n nodal values were obtained. The network's weights and biases are $w_{ij}$ and ${\;b}_{j}$.

(24) $$y_{j}=f\left(\sum_{i=1}^{n}w_{ij}x_{i}+b_{j}\right)$$

Neural networks are frequently trained utilizing backpropagation (BP) methods. The Levenberg-Marquardt algorithm was used as the BP algorithm in this research work because it is frequently the fastest BP algorithm in training [83].

### 7.4 Gaussian process regression

The Gaussian process regression **(**GPR) is a powerful generalization tool used in high-dimensional and nonlinear regression situations. It is a probabilistic prediction method created using statistical learning and Bayesian theory. Some random variables with a multivariable Gaussian distribution make up a Gaussian process (GP). Utilizing the mean and covariance functions of the input features, it predicts results. It makes the assumption that the noise is additive, stationary, and has a Gaussian distribution. To calculate the output, the GPR model takes values from the input and a latent variable. A priori Gaussian assumption and interpolation capabilities are two of the GPR's benefits. The correlation between inputs is ascertained by the covariance function, whereas the mean function is employed when the input space is missing or unobserved. Given the importance of the covariance function in the GPR model, it is extremely important to carefully select an appropriate covariance function and the variables that govern it before beginning the process of training GPR models. The geometric structure of the training samples is embedded by this function. In other words, the mean and covariance functions, also known as hyper parameters, should be estimated from the used data in order to produce precise predictions [84]. GPR is discussed in more technical detail in Refs. [[85], [86], [87], [88]].

## 8 Results and discussion of ML models

### 8.1 Hyper-parametric configurations

In this study, XGB, RF, ANN, and GPR models were constructed to estimate the confined axial load-carrying capacity, axial strain, and lateral strain. To establish the hyper parameter, architectures, and functions of the models, a trial-and-error tuning method was carried out throughout the training phase. In this study, to implement the ANN and GPR models, MATLAB 2018a software was used. There are five input nodes, one output node, and nine hidden-layer nodes in the ANN architecture for output 1. Whereas for output 2 and output 3, there are five input nodes, one output node, and eleven hidden-layer nodes. Python has been used for the XGB and RF models, which is most widely employed language in data science. The libraries of Pandas, Keras, Tensorflow, and Scikit learn were imported and used to train the models. The optimal XGB and RF hyper-parameters and architectures are summarized in Table 11. Using the trial-and-error process, the GPR model was tunned, leading to find two crucial parameters: the width of the radial basis function as 0.38, 0.17, and 0.23, and the Gaussian noise of 0.05, 0.3, and 0.21, respectively, for output 1, output 2, and output 3.

**Table 11:** Optimized hyper-parameters of ensemble models.

	Hyper-parameter	Optimized value		
		Output 1	Output 2	Output 3
XGB	Estimators	105	100	90
	Learning rate	0.3	0.6	0.5
	Max depth	10	14	12
RF	Estimators	90	90	110
	Min samples split	2	2	2
	Max features	3	3	2
	Max depth	20	18	20

### 8.2 Details of performance indices

Six distinct performance metrics were calculated to evaluate the performance of the employed models [70,[89], [90], [91]]. The ideal values and parametric equations are presented in Table 12. For a perfect prediction model, these parameters' values must exactly match their ideal values.

**Table 12:** Performance metrics equation and their ideal values.

No.	Parameter	Equation	Ideal value
1	Coefficient of determination (*R*^*2*^)	$R^{2}=\frac{\sum_{i=1}^{n}{\left(y_{i}-y_{mean}\right)}^{2}-\sum_{i=1}^{n}{\left(y_{i}-{\hat{y}}_{i}\right)}^{2}}{\sum_{i=1}^{n}{\left(y_{i}-y_{mean}\right)}^{2}}$ where $y_{i}$ and ${\hat{y}}_{i}$ are actual and predicted $i^{\text{th}}$ values, respectively.	1
2	Adjusted determination coefficient (adj.*R*^*2*^)	adj$.\;R^{2}=1-\frac{\left(n-1\right)}{\left(n-p-1\right)}\left(1-R^{2}\right)$ where *p* is number of input parameters.	1
3	Variance account factor (VAF)	VAF $=\left(1-\frac{var\left(y_{i}-{\hat{y}}_{i}\right)}{var\left(y_{i}\right)}\right)\times 100$	100
4	Performance index (PI)	PI = adj.$R^{2}$ + 0.01VAF – RMSE	2
5	Root mean square error (RMSE)	RMSE = $\sqrt{\frac{1}{N}\sum_{i=1}^{n}{\left(y_{i}-{\hat{y}}_{i}\right)}^{2}}$ N is number of data sample.	0
6	Mean absolute error (MAE)	MAE $=\frac{1}{n}\sum_{i=1}^{m}\left|\left({\hat{y}}_{i}-y_{i}\right)\right|$	0

### 8.3 Statistical details of results

The models constructed to estimate the three outputs using the five input variables are discussed in this section along with their respective predictive results. Table 13 detail the models' performance at predicting the training and testing outputs. It is essential to point out that the evaluation of how well each model performed on the data used for training was the factor that was utilized to quantify the level of goodness of fit. Results of the experiment illustrated that for output 1 (confined axial load-carrying capacity), the XGB model had the highest *R*^*2*^ and lowest RMSE values during training (*R*^*2*^ = 0.9969 and RMSE = 0.0156). The testing results for the XGB's predictive ability were *R*^*2*^ = 0.9817 and RMSE = 0.0357. In terms of the prediction accuracy, the RF model tied with the XGB model, while the ANN and GPR models both fell slightly short. Similarly, for outputs 2 and 3 (the confined axial strain and confined lateral strain, respectively), the XGB model obtained the highest desired accuracy among the developed ML models during both training and testing. In the testing phase, the RMSE values of the XGB model were 0.0058 (output 2) and 0.0377 (output 3), and the *R*^*2*^ values were 0.9992 (output 2) and 0.9485 (output 3). For output 2, the RF was the second-best model in both phases. The ANN model was the worst model in training (*R*^*2*^ = 0.6936), whereas it performed well compared to the GPR model in testing (*R*^*2*^ = 0.6845).

**Table 13:** Accuracy evaluation of models' performance in training and testing.

Training							
		***R***^***2***^	**Adj.*R***^***2***^	**VAF**	**PI**	**RMSE**	**MAE**
**Output 1**	XGB	0.9969	0.9966	99.698	1.9780	0.0156	0.0050
	RF	0.9921	0.9913	99.1140	1.9556	0.0268	0.0190
	ANN	0.9608	0.9569	95.8445	1.8558	0.0596	0.0445
	GPR	0.9712	0.9684	97.1164	1.8913	0.0482	0.0365
**Output 2**	XGB	0.9984	0.9982	99.8412	1.9883	0.0083	0.0040
	RF	0.9411	0.9353	93.5500	1.8176	0.0532	0.0308
	ANN	0.6936	0.6635	68.3031	1.2217	0.1248	0.0832
	GPR	0.8386	0.8227	80.9897	1.5413	0.0913	0.0637
**Output 3**	XGB	0.9101	0.9013	91.0173	1.7587	0.0527	0.0231
	RF	0.8328	0.8164	80.7370	1.5465	0.0773	0.0581
	ANN	0.5881	0.5477	58.0558	1.0131	0.1151	0.0856
	GPR	0.5862	0.5456	49.6075	0.9167	0.1250	0.0913

Testing							
		***R***^***2***^	**Adj.*R***^***2***^	**VAF**	**PI**	**RMSE**	**MAE**
**Output 1**	XGB	0.9817	0.9747	98.1757	1.9207	0.0357	0.0151
	RF	0.9723	0.9617	97.1931	1.8893	0.0442	0.0314
	ANN	0.9451	0.9239	94.2142	1.7959	0.0701	0.0607
	GPR	0.9376	0.9136	93.7605	1.7852	0.0659	0.0543
**Output 2**	XGB	0.9992	0.9989	99.9193	1.9922	0.0058	0.0039
	RF	0.9879	0.9832	98.1363	1.9364	0.0281	0.0202
	ANN	0.7322	0.6293	72.8552	1.2468	0.1109	0.0776
	GPR	0.6845	0.5632	63.2768	1.0730	0.1230	0.0893
**Output 3**	XGB	0.9485	0.9287	94.8546	1.8395	0.0377	0.0218
	RF	0.9247	0.8957	90.9915	1.7554	0.0502	0.0390
	ANN	0.4908	0.2950	47.8583	0.6501	0.1235	0.1140
	GPR	0.4035	0.1741	36.2184	0.4036	0.1326	0.1055

**Fig. 16:**
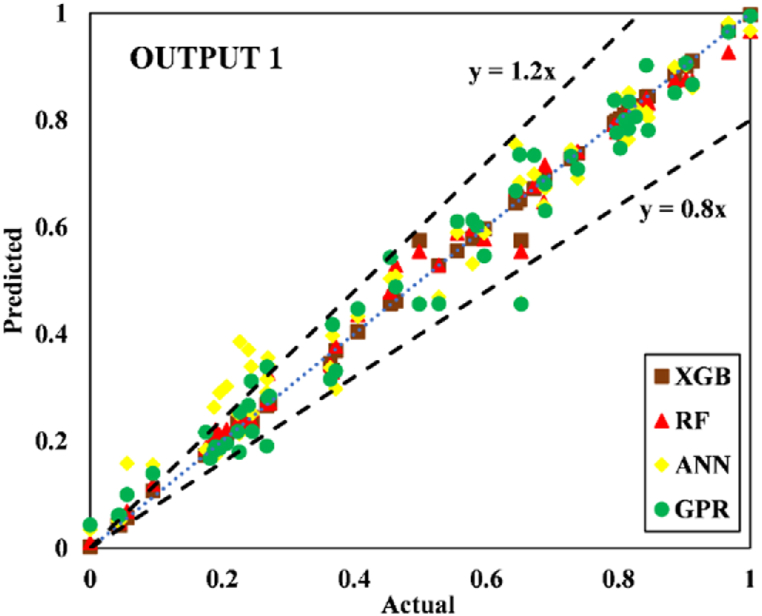
Scatter plot for output 1 in training.

**Fig. 17:**
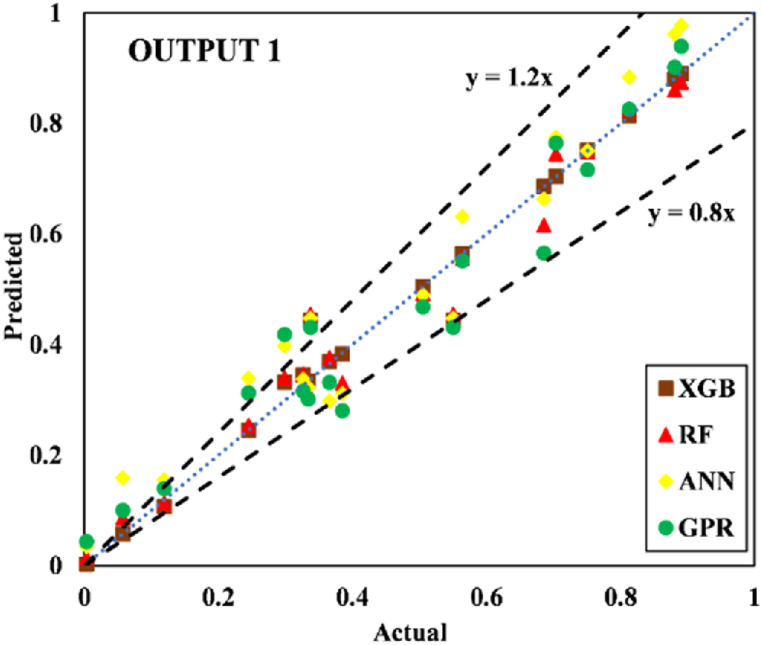
Scatter plot for output 1 in testing.

**Fig. 18:**
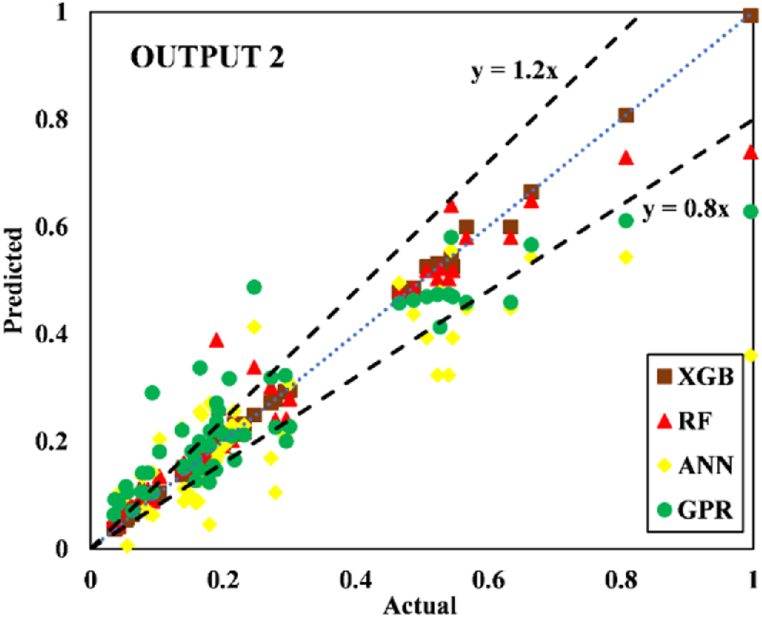
Scatter plot for output 2 in training.

**Fig. 19:**
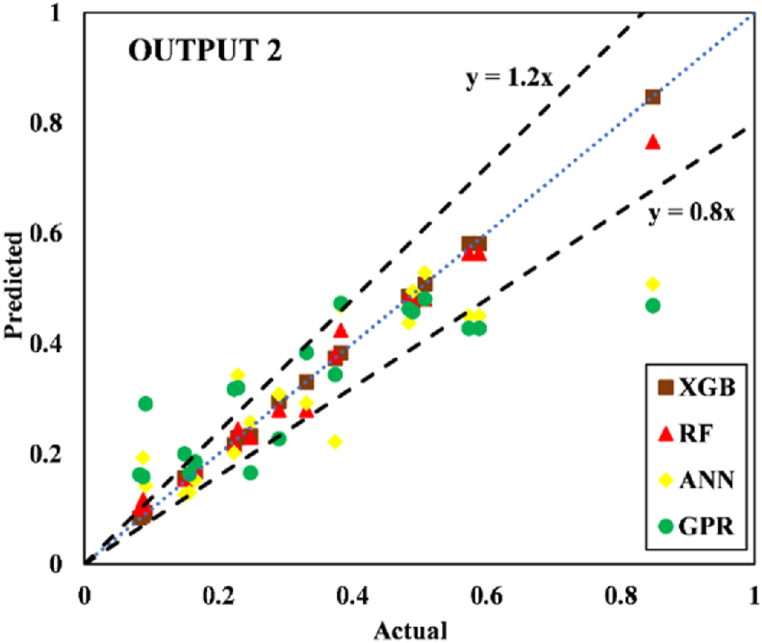
Scatter plot for output 2 in testing.

**Fig. 20:**
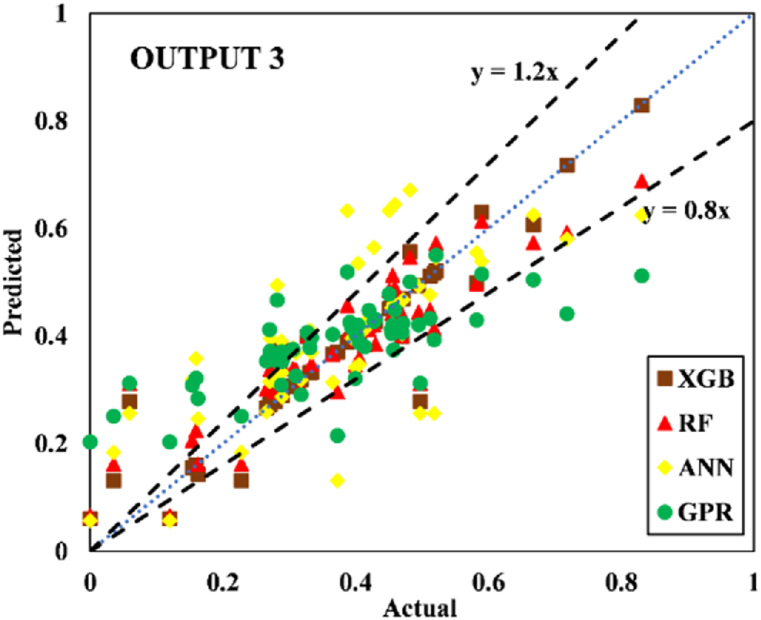
Scatter plot for output 3 in training.

**Fig. 21:**
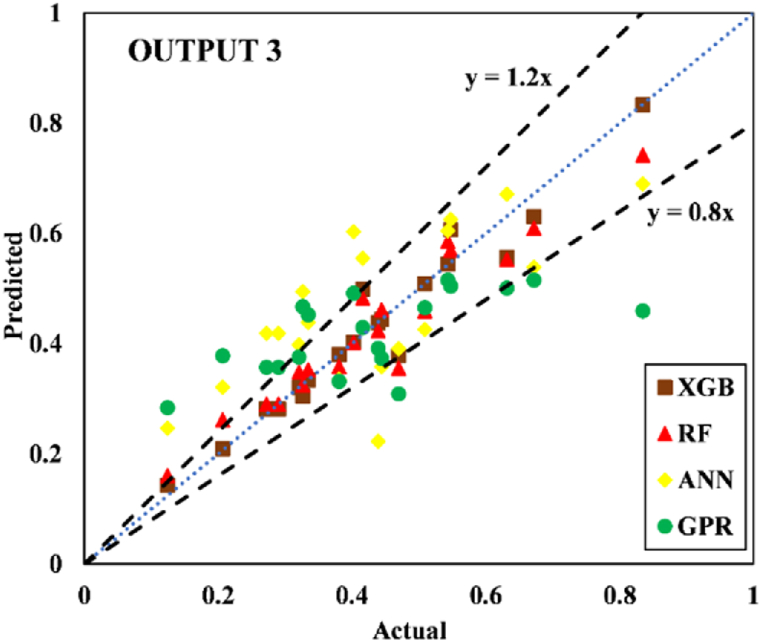
Scatter plot for output 3 in testing.

For output 3, the XGB and RF models had reduced predictive performance across both prediction phases compared to its performance for output 1 and output 2. The ANN and GPR models, whose performance was good for output 1, failed to perform well for output 2 and output 3. In fact, for output 3, they obtained an *R*^*2*^ value even lower than 0.50 in testing, indicating its inability to predict output 3. Out of the models proposed for the scenario with three outputs, Table 13 shows that XGB performed best in both prediction phases. The three outputs are depicted on the regression plots in Fig. 16, Fig. 17, Fig. 18, Fig. 19, Fig. 20, Fig. 21 for the training and testing phases of the prediction models.

The relative importance in percentage of the input parameters as input 1, input 2, input 3, input 4, and input 5 obtained by the RF method for predicting output 1 were 17.0866, 1.859382, 2.067616, 4.900994, and 74.0853, respectively, implying the dominance of input 5 in prediction. The relative importance of input 1, input 2, input 3, input 4, and input 5 for output 2 was 21.291576, 14.439464, 14.860123, 25.781864, and 23.62697, respectively, whereas for output 3 they were 13.8679, 17.759356, 10.172258, 23.2137, and 34.986785, respectively. For output 2 and output 3, all the input variables had a significant contribution to the prediction.

**Fig. 22:**
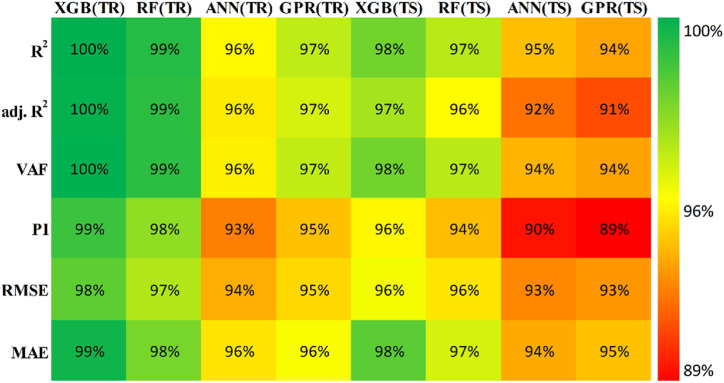
Accuracy matrix for output 1 in training and testing phases.

**Fig. 23:**
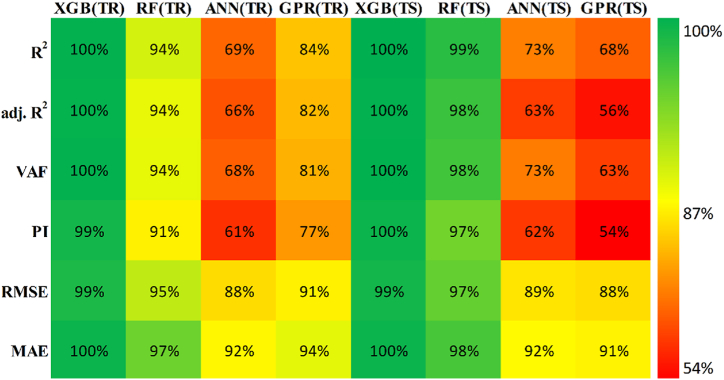
Accuracy matrix for output 2 in training and testing phases.

**Fig. 24:**
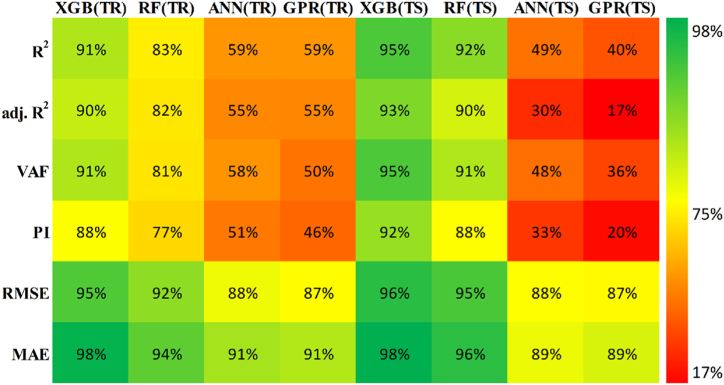
Accuracy matrix for output 3 in training and testing phases.

Researchers frequently rely on the accuracy matrix, which is a heat map matrix that was developed relatively recently. This matrix was developed to assess the performance of a model across a variety of metrics. Without checking the values of the metrics, the accuracy matrix allows one to quickly ascertain the level of accuracy achieved by a model [91,92]. Fig. 22, Fig. 23, Fig. 24 display the accuracy matrix of the developed models for predicting the confined axial load-carrying capacity (kN), confined axial strain (mm/mm), and confined lateral strain (mm/mm), respectively,. It is evident from the accuracy matrix that for output 1, the accuracy of all four predictive models (XGB, RF, ANN, and GPR) was very good. For output 2, XGB and RF had good accuracy, but ANN and GPR had slightly decreased accuracy. For output 3, ANN and GPR were very inaccurate, as can be seen from Fig. 24. By assessing the overall performance of the developed models, it can be concluded that out of the proposed four models, XGB and RF can be used to predict results for all three outputs, whereas ANN and GPR can be utilized to predict output 1 and output 2 satisfactorily.

## 9 Conclusions

The parametric study on the structural response of rectangular FRP-confined concrete columns reinforced by high-strength elliptical steel tube was presented in this article. The effects of the variables such as the strength of concrete and thickness and cross-sectional size of the steel tube on the response of rectangular DSTCCs were investigated using nonlinear FEA. Furthermore, four different techniques of ML models proposed for predicting the axial load, axial strain, and lateral strain of DSTCCs were discussed. From the findings of the study, the following conclusions were drawn.

• FEM developed for this investigation is capable of replicating the experimental tests in predicting the structural response of rectangular DSTCCs under concentric axial load.
• The confined concrete strength enhancement ratio decreased as the strength of the confined concrete increased. The lower concrete strength confined in DSTCCs gained more strength from confinement in proportion to its unconfined strength than higher-strength concrete.
• Increasing the concrete strength improved the stiffness in the elastic range and the load-carrying capacity of DSTCCs. It was observed that the stiffness of DSTCCs in the elastic range with lower concrete grade could be enhanced by increasing the confining CFRP thickness.
• The ultimate strain of DSTCCs before the onset of the CFRP tensile rupture could be enhanced by increasing the aspect ratio and the confining CFRP thickness. DSTCCs with low unconfined concrete strength and aspect ratios 2.0–2.5 exhibited large deformation before the CFRP rupture.
• The axial load-carrying capacity of DSTCCs was substantially boosted by increasing the thickness of the inner elliptical steel tube.
• The thicker elliptical steel tube in DSTCCs effectively improved the strength and ultimate strain of the confined concrete compared to DSTCCs with the thinner elliptical steel tube having the same aspect ratio and CFRP thickness.
• The cross-sectional size of the inner elliptical steel tube had no considerable influence on the response of DSTCCs. By increasing the cross-sectional size of the inner elliptical steel tube, the axial load-carrying capacity and confined strain of DSTCCs improved.
• Based on the statistical performance metrics, the XGB, RF, ANN, and GPR models predicted the ultimate load-carrying capacity of DSTCCs with good accuracy. In predicting the axial and lateral strains, the XGB and RF models demonstrated better performance than the AAN and GPR models.

## 10 Data availability statement

All data used in this investigation are presented in the article.

## Funding

This research received no external funding.

## Additional information

No additional information is available for this article.

## CRediT authorship contribution statement

**Haytham F. Isleem:** Conceptualization, Data curation, Formal analysis, Investigation, Validation, Methodology, Project administration, Resources, Software, Supervision, Visualization, Writing – original draft. **Besukal Befikadu Zewudie:** Conceptualization, Data curation, Formal analysis, Investigation, Validation, Resources, Software, Visualization, Writing – original draft, Writing – review & editing. **Alireza Bahrami:** Conceptualization, Data curation, Formal analysis, Investigation, Validation, Methodology, Project administration, Resources, Software, Supervision, Visualization, Writing – original draft, Writing – review & editing. **Rakesh Kumar:** Conceptualization, Data curation, Formal analysis, Investigation, Validation, Methodology, Resources, Software, Visualization, Writing – original draft, Writing – review & editing. **Wang Xingchong:** Software, Visualization, Writing – review & editing. **Pijush Samui:** Project administration, Resources, Software, Visualization, Writing – review & editing.

## Declaration of competing interest

The authors declare that the research was conducted in the absence of any commercial or financial relationships that could be construed as a potential conflict of interest.
